# Outstanding Antibacterial Activity of *Hypericum rochelii*—Comparison of the Antimicrobial Effects of Extracts and Fractions from Four *Hypericum* Species Growing in Bulgaria with a Focus on Prenylated Phloroglucinols

**DOI:** 10.3390/life13020274

**Published:** 2023-01-18

**Authors:** Yana Ilieva, Teodor Marinov, Iliyan Trayanov, Mila Kaleva, Maya Margaritova Zaharieva, Lyubomira Yocheva, Zlatina Kokanova-Nedialkova, Hristo Najdenski, Paraskev Nedialkov

**Affiliations:** 1 Department of Infectious Microbiology, The Stephan Angeloff Institute of Microbiology, Bulgarian Academy of Sciences, 1113 Sofia, Bulgaria; 2 Pharmacognosy Department, Faculty of Pharmacy, Medical University of Sofia, 1000 Sofia, Bulgaria; 3 Department of Chemical Engineering, Faculty of Chemical and System Engineering, University of Chemical Technology and Metallurgy, 1756 Sofia, Bulgaria; 4 Institute of Chemical Engineering, Bulgarian Academy of Sciences, 1113 Sofia, Bulgaria; 5 Department of Biology, Medical Genetics and Microbiology, Faculty of Medicine, Sofia University “St. Kliment Ohridski”, 1407 Sofia, Bulgaria

**Keywords:** *Hypericum*, extracts, phytochemicals, microbes, *S. aureus*, MRSA, bacterial biofilm

## Abstract

Microbial infections are by no means a health problem from a past era due to the increasing antimicrobial resistance of infectious strains. Medicine is in constant need of new drugs and, recently, plant products have had a deserved renaissance and garnered scientific recognition. The aim of this work was to assess the antimicrobial activity of ten active ingredients from four *Hypericum* species growing in Bulgaria, as well as to obtain preliminary data on the phytochemical composition of the most promising samples. Extracts and fractions from *H. rochelii* Griseb. ex Schenk, *H. hirsutum* L., *H. barbatum* Jacq. and *H. rumeliacum* Boiss. obtained with conventional or supercritical CO_2_ extraction were tested on a panel of pathogenic microorganisms using broth microdilution, agar plates, dehydrogenase activity and biofilm assays. The panel of samples showed from weak to extraordinary antibacterial effects. Three of them (from *H. rochelii* and *H. hirsutum*) had minimum inhibitory concentrations as low as 0.625–78 mg/L and minimum bactericidal concentrations of 19.5–625 mg/L against *Staphylococcus aureus* and other Gram-positive bacteria. These values placed these samples among the best antibacterial extracts from the *Hypericum* genus. Some of the agents also demonstrated very high antibiofilm activity against methicillin-resistant *S. aureus*. Ultra-high-performance liquid chromatography–high-resolution mass spectrometry revealed the three most potent samples as rich sources of biologically active phloroglucinols. They were shown to be good drug or nutraceutical candidates, presumably without some of the side effects of conventional antibiotics.

## 1. Introduction

Antimicrobial resistance (AMR) in numerous bacterial species is a well-known health threat and medicinal challenge. Annual global deaths due to this phenomenon have risen to approximately 750,000, and are projected to reach as high as 10 million by the year 2050 [1]. The overuse of antibiotics is one of the main reasons for the rise in the selection for AMR [2]. A postantibiotic era, in which simple infections and minor injuries can kill, is far from an apocalyptic fantasy, but a very real possibility for the 21st century [3].

Over the past 20 years, *Staphylococcus aureus* infections have become more dangerous and more expensive to treat due to the increasing prevalence of AMR in the species [3]. Currently, methicillin-resistant *S. aureus* (MRSA) has become the main type of *S. aureus* infection and, thus, one of the main human pathogens [4]. Its isolates are more frequently associated with mortality than infections caused by other bacteria [3]. MRSA responds to some current antibiotics, but these effects may not last long due to the constant mutations of this strain [5]. Bacterial biofilms contribute to >80% of all infections in humans [3]. MRSA is biofilm forming and, together with *P. aeruginosa*, is one of the most ubiquitous pathogens in biofilms found in healthcare [6]. The MRSA biofilm causes not only persistent infections and colonization on catheters and other devices, but also significant mortality in patients with wounds and necrotic tissue [3,4,7,8].

Due to the increased AMR towards established antibiotics, the use of medicinal plants has become desirable, and has been receiving rising attention over the past few decades. There are many published reports on traditional herbs and natural products of higher plants. Besides oncology and immunoregulation, therapeutic effects of natural product-derived drugs are predominantly achieved in antibiotic therapies [9]. They are effective against many bacteria and infectious diseases, while simultaneously mitigating many of the side effects of conventional antimicrobials [10,11]. Moreover, natural products have an economic advantage: they could be used to fuel future discovery pipelines, since the cost of bringing a new antibiotic from discovery to market is high, return on investment is low and the development of new antibiotics has slowed dramatically since the 1950s’ golden age of discovery [12].

*Hypericum* L., or St John’s wort (*Hypericaceae*), is a genus of grasses and shrubs of more than 480 species found in all temperate parts worldwide. *H. perforatum* L., the most prominent and recognized *Hypericum* species, is an approved drug for depression. In addition, it and other *Hypericum* plants have been used in traditional medicine as antimicrobials for external use—for example, for infected wounds [13]. This is related to the pronounced antimicrobial effect [14] mainly of their major secondary metabolites, the phloroglucinols, e.g., polycyclic polyprenylated acylphloroglucinols (PPAPs) such as hyperforin, the main antibacterial principle in *H. perforatum* [15,16,17,18,19,20,21,22]. In some cases, naphtodianthrones (hypericins) also exert an antibacterial effect [5,23,24], as well as benzopyrans [25], xanthones [26], etc.

*H. perforatum* has a well-established antibacterial effect [24,27,28]. In addition to depression, it has been clinically used to treat infections—preparations involving acetone, going by the name novoimanine, have been used in Russia [22,28,29]. Extracts of the same plant have been patented in the United States as a food preservative [30].

The antimicrobial effect has been found in extracts and essential oils [31] from numerous other St John’s wort species (spp.), and has been diligently described in reviews [24,32,33,34], e.g., *H. japonicum* Thunb. [35,36], *H. brasiliense* Choisy [37], *H. calycinum* L. [38], *H. havvae* A. Guner [39], etc. [40,41,42,43,44].

The extracts and phytochemicals from *Hypericum* spp. affect primarily, but not exclusively [24,45,46], Gram-positive bacteria [23,27,33,47,48], possibly due to the outer membrane of Gram-negative bacteria.

Generally, the two main methods to assess the antimicrobial effect are through broth (micro)dilution, which determines a minimum inhibitory concentration (MIC), and the disc diffusion method, which gives a zone of bacterial inhibition. Different St John’s wort spp. have a broad range of antibacterial activity. MICs of *Hypericum* extracts can be as low as 5 mg/L or as high as 2500 mg/L [28,49], usually tens or hundreds of mg/L. To the best of our knowledge, the most potent extract belonged to a hydroalcoholic extract of *H. perforatum* with a MIC of 0.625 mg/L against cariogenic *Lactobacillus* spp. [50].

The aim of the present study was to test ten active ingredients (extracts and fractions) from four *Hypericum* spp. (*H. rochelii* Griseb. ex Schenk, *H. hirsutum* L., *H. barbatum* Jacq. and *H. rumeliacum* Boiss.) growing in Bulgaria for antimicrobial and antibiofilm activity on a panel of pathogenic microorganisms. Additionally, we aimed to obtain insight into the chromatographic profile of the most promising samples with ultra-high-performance liquid chromatography–high-resolution mass spectrometry (UHPLC–HRMS).

The panel of pathogenic microorganisms involved *S. aureus*, MRSA and *Pseudomonas aeruginosa*, which are included in the ESKAPE acronym (*Enterococcus faecium*, *S. aureus*, *Klebsiella pneumoniae*, *Acinetobacter baumannii*, *P. aeruginosa* and *Enterobacter* spp.). This group was accepted to comprise highly virulent bacteria with increasing AMR, the major cause of life-threatening nosocomial infections in immunocompromised and critically ill patients [51].

## 2. Materials and Methods

### 2.1. Plant Material Collection and Preparation

Aerial parts of *H. barbatum* Jacq. (voucher no. 177790) were collected from the Konyavska mountain, Bulgaria, in June 2021. Aerial parts of *H. rumeliacum* Boiss (voucher no. 177787) were collected from the Bela voda, Pernik and Konyavska mountain, Bulgaria, in June 2021. Aerial parts of *H. rochelii* Griseb. et Schenk (voucher no. 177786) were collected from Lakatnik rocks, Lakatnik, Bulgaria, in June 2021. Aerial parts of *H. hirsutum* L. (voucher no. 177784) were collected from Uzana, the Stara Planina mountain, Bulgaria, in July 2021. All plant materials were gathered during the flowering period. The plants were identified by P. Nedialkov. Voucher specimens were deposited at the National Herbarium, the Bulgarian Academy of Sciences, Sofia, Bulgaria. All plant materials were air-dried in the shade and powdered. Afterwards, they were sieved through a sieve with a pore size of 0.3 mm, so that the largest particles were no bigger than 0.3 mm in size.

### 2.2. Extraction

#### 2.2.1. Supercritical CO_2_ Extraction

The extraction was performed with a Supercritical Fluid Extractor SFT-100XW with SFT-25 SCF Pump (CP/CF Version), which provided a flow rate of up to 125 mL/min and a pressure of up to 690 bar, as well as a heater (Supercritical Fluid Technologies Inc., Newark, DE, USA). For safety reasons, the air in the laboratory was ventilated well during experiments, thus, the high CO_2_ output. The conditions for the extraction of the plant materials were guided with the authors’ previous experience in supercritical fluid extraction and using the protocol of Römpp et al., which was associated with high selectivity and enrichment for phloroglucinols [52]. The conditions were as follows: 120 bar extraction pressure, 38 °C extraction temperature, 44 °C temperature at the input valve, flow rate 0.74862 L/min (1 L/min when draining). First, the powdered aerial parts (5.3661 g) were extracted with CO_2_ only and then the same materials continued to be extracted with CO_2_ and modifier methanol (MeOH) (10%) as the coextractor to yield two extracts, RochC (dry) and RochCM (liquid), respectively. The extraction was carried out through several soakings (each with a duration of approximately 20 min) and drainings (each with a duration of approximately 40 min), determined and guided using the yield after each draining until the total depletion of plant materials. In the end of both extractions, the plant mass was removed and MeOH was used to wash its leftovers from the system.

#### 2.2.2. Conventional Extraction

The powdered aerial parts of *H. rochelii* (22.0506 g) were, subsequently, extracted with dichloromethane (CH_2_Cl_2_) (12 × 100 mL), MeOH (4 × 150 mL) and 80% aq. MeOH (2×100 mL). The resulting extracts were evaporated to dryness using a vacuum rotary evaporator. The CH_2_Cl_2_ extract gave a dark green waxy residue of 1.403 g (RochD). The MeOH and aq. MeOH residues were combined to give 4.4453 g of a brown semisolid (RochM).

The powdered aerial parts of *H. hirsutum* (412.0457 g) were extracted with CH_2_Cl_2_ (36 × 500 mL) at room temperature. The CH_2_Cl_2_ extract gave a dark green waxy residue of 40.8 g. The CH_2_Cl_2_ extract was subjected to column chromatography (CC) over a Diaion HP-20 (5 × 15 mm) and was, subsequently, eluted with 90% aq. MeOH (15 × 500 mL) and MeOH (5 × 500 mL) to obtain 2 pooled fractions of 24.1 g (HirDM90) and 5.1 g (HirDM100), respectively. An elution with CH_2_Cl_2_ gave a fraction containing lipids, chlorophyll and waxes (HirDD).

Powdered aerial parts of *H. barbatum* (12.102 g), *H. rumeliacum* (Bela voda, Pernik) (10.4469 g) and *H. rumeliacum* (Konyavska mountain) (11.4158 g) were extracted separately with CH_2_Cl_2_ (10 × 200 mL), combined and then evaporated in vacuo to give dark green waxy residues of 863.5 mg (BarbD), 539.4 mg (RumDBe) and 582 mg (RumDKo), respectively.

### 2.3. Ultra-High-Performance Liquid Chromatography–High-Resolution Mass Spectrometry Conditions

The UHPLC–HRMS analysis was performed using a Thermo Scientific Dionex Ultimate 3000 RSLC (Germering, Germany) consisting of a 6-channel degasser SRD-3600, high-pressure gradient pump HPG-3400RS, autosampler WPS-3000TRS and column compartment TCC-3000RS coupled to a Thermo Scientific Q Exactive Plus (Bremen, Germany) mass spectrometer. The sample solution for injection was prepared by dissolving samples in MeOH, diluting to a concentration of ca. 20 µg/mL with MeOH and filtering through a 0.22 µm syringe PVDF membrane filter. UHPLC separations were performed on a Waters CORTECS C18 column (2.1 × 100 mm, 90 Å, 2.7 μm) equipped with a precolumn Waters CORTECS C18 VanGuard at 40 °C. Each chromatographic run was carried out with a binary mobile phase consisting of water containing 0.1% (*v*/*v*) formic acid (A) and acetonitrile, also with 0.1% (*v*/*v*) formic acid (B). A gradient program was used as follows: 0–1 min, 50% B; 1–22 min, 50–80% B; 22–25 min, 80% B; 25–27 min, 80–85% B; 27–30 min, 85% B; 30–32.5 min, 85–95% B; and 32.5–34.5 min, 95% B. The system was equilibrated with the initial conditions for 4.5 min. The solvent flow rate and the sample injection volume were 300 μL min^−1^ and 2 µL, respectively. The operating conditions for the HESI source used in a positive ionization mode were a +3.5 kV spray voltage, 320 °C capillary and probe heater temperature, sheath gas flow rate of 36 a.u., auxiliary gas flow of 11 a.u. (a.u. refers to arbitrary values set with the Exactive Tune software) and S-Lens RF level of 50.00. Nitrogen was used for sample nebulization and collision gas in the HCD cell. Top5 was used as an MS experiment, where for the full scans, the resolution, AGC target, max. IT and mass range were set to 70,000 (at *m*/*z* 200), 3 × 10^6^, 100 ms and *m*/*z* 150–1000, respectively. While the ddMS^2^ conditions were set to a resolution of 17,500 (at *m*/*z* 200), the AGC target was 1 × 10^5^, max. IT was 50 ms, the isolation window was 2.0 *m*/*z* and NCE was 15. For the quantitative determination of the main constituents of the samples with the highest antibacterial effect (RochC, RochD and HirDM90, the most active samples) only a full-scan MS experiment was used with the following parameters: the resolution, AGC target, max. IT and mass range were set to 70,000 (at *m*/*z* 200), 3 × 10^6^, 200 ms and *m*/*z* 300–600, respectively. The contents of the constituents were calculated as methoxyhyperibine J [53]. Xcalibur software ver. 4.0 was used for data acquisition and processing.

### 2.4. Bacterial Strains and Growth Conditions

The panel of pathogenic microorganisms used in this study consisted of *Staphylococcus aureus* (American Type Cell Culture Collection, (ATCC) 29213, Manassas, VA, USA), *Staphylococcus aureus*–MRSA, resistant to methicillin and oxacillin (#1337, Collection of the Stephan Angeloff Institute of Microbiology, Sofia, Bulgaria (SAIMC)), *Enterococcus faecalis* (ATCC 29212), *Streptococcus pyogenes* (SAIMC 10535, Collection of SAIMC), as well as the Gram-negative *Escherichia coli* (ATCC 35218), *Pseudomonas aeruginosa* (ATCC 27853), *Yersinia enterocolitica* (1WA8081 0:8) and the yeast *Candida albicans* (CBS 562, The Westerdijk Institute, Utrecht, The Netherlands). Bacteria were maintained in tryptone soya broth (TSB) (LQ009, Himedia, Mumbai, India) at 37 °C, aerobic conditions. For the experiments, Mueller–Hinton broth (MHB), (GM391, Himedia, Mumbai, India) was used for *E. coli* and *S. aureus*, while brain heart infusion (BHI) broth (M210, Himedia, Mumbai, India) was used for all other strains.

### 2.5. Determination of Minimum Inhibitory Concentration (MIC) with Broth Microdilution Method (BMD)

The BMD was carried out according to ISO 20776/1-2006 [54] as follows: a bacterial suspension with a density of 10^8^ CFU/mL (0.5 McFarland standard, OD_600_) was prepared from an overnight grown liquid bacterial culture brought to a working bacterial suspension (WBS) with a concentration of 5 × 10^5^ CFU/mL by being diluted 200× with MHB or BHI broth and homogenized over a vortex. The tested extracts (stock solutions in ethanol) were prepared in two-fold serial dilutions in 96-well round-bottom plates to a volume of 50 µL. The concentrations usually ranged from 5000 to 9.8 or 4.9 mg/L, as can be seen in the figures for the bacterial metabolic activity in Results, or to lower values, in order to obtain MICs or concentrations of the inhibition of colony-forming units (CFUs). The MHB or BHI broths were used as diluents and as tests for the absence of contamination. At least 8 wells were left for control and blank probes. An equivalent volume (50 μL) of the WBS was inoculated in each well on the plates, except the blank wells, achieving a final bacterial density of 5 × 10^4^ CFU/mL. After 18–24 h incubation under aerobic standardized conditions at 37 °C, the plates were examined. The growth in the positive control wells was checked for sufficient growth and the MIC was determined manually as the lowest concentration that completely prevented or inhibited visible bacterial growth, as detected with the unaided eye. In the end, the plates that contained active agents were subjected to an assessment of the dehydrogenase activity of the bacteria. For the reference antibiotics, we used gentamicin (GEN, #15750-037, 50 mg/mL, Gibco, Paisley, United Kingdom) for *S. aureus*, MRSA, *E. faecalis*, *E. coli* and *P. aeruginosa*; penicillin (PEN, #B0500000, Merck KGaA, Darmstadt, Germany) for *E. faecalis* and *S. pyogenes*; ciprofloxacin (CIP, Ciproflav:10 mg/mL, Polfa S.A.Warsaw Pharmaceutical Works, Starogard Gdanski, Poland) for *Y. enterocolitica* and for *E. faecalis*, which is resistant to penicillin and gentamicin; and amphotericin B (AMP, #AMP-B, Capricorn Scientific GmbH, Ebsdorf, Hessen, Germany) for *C. albicans*. The ranges of concentrations used were 0.02–8 mg/L (GEN), 0.004–4 mg/L (PEN), 0.005–2 mg/L (CIP) and 0.02–2 mg/L (AMP).

### 2.6. Assessment of the Cell Redox (Dehydrogenase or Metabolic) Activity

Dehydrogenase activity was assessed according to the protocol of Wang et al. [55], with minor modifications. MTT dye was used, since it is reduced by the membrane-located bacterial enzyme NADH, ubiquinone reductase, to formazan crystals. Ten microliters of a 5 mg/mL MTT solution in phosphate-buffered saline (PBS) were added to each well at the end of the incubation period after reading the BMD test and homogenization. The plate was incubated at the same conditions for 1 h (or more if the strains had weak growth and dehydrogenase activity). Then, the formazan crystals were dissolved using an equivalent volume (100 μL/well) of a 5% formic acid solution in 2-propanol. The absorbance was measured at 550 nm (Absorbance Microplate Reader Lx800, Bio-Tek Instruments Inc., Winooski, VT, USA) with the lid against a blank solution containing respective volumes of broth, MTT and solvent. Dehydrogenase activity was calculated as a percentage of the activity of the normalized control.

### 2.7. Determination of Minimum Bactericidal Concentration (MBC) and Concentrations of Inhibition of Colony-Forming Unit (CFU) Growth with Agar Plate Assay (APA)

An APA was performed using a modified protocol of Mah [56], as described in [57]. The broths used for the BMD were subcultured onto fresh agar plates. Wells from the BMD, which had not been used for the dehydrogenase measurement, were used with MIC and remaining higher concentrations of the extracts. Petri dishes with the BHA agar (M211, Himedia, Mumbai, India) were divided into 8 squares, and in each one, 10 µL of a sample was dropped. After drying, the dishes were turned with their lids down and cultivated under aerobic standardized conditions at 37 °C for 16–24 h and examined. A concentration was accepted to inhibit the CFU growth if it resulted in single colonies or at least 50% countable colonies. That value was usually higher than the MIC. The MBC was the lowest concentration where no growth was observed.

### 2.8. Biofilm Assay of MRSA

For this assay, we chose samples with different activities (RochD, HirDM90, HirDM100 and RumDKo). Equieffective concentrations of the MIC—MIC, 1/2 MIC, 1/4 MIC, 1/8 MIC and 1/16 MIC and, where the MIC could not be determined (RochD) 10, 5, 2.5, 1.25 and 0.625 mg/L—were prepared in 96-well polystyrene flat-bottom tissue culture plates in BHI containing 2% glucose (*w*/*v*) to a final volume of 100 µL/well. The bacterial inoculum of MRSA was prepared, added in equivalent volumes (100 µL) and incubated in the same way as described above for the BMD protocol. Next, an optimized protocol for the visualization of the biofilms [58] was applied. First, the supernatant containing planktonic cells was removed and the wells were washed carefully 3× with 200 μL/well PBS. Then, remaining attached cells were fixed through incubation with 200 μL/well methanol for 15 min at room temperature. After that, the methanol was removed and the plate was dried with air for 5–10 min. Fifty microliters of 2% Hucker crystal violet were added to each well and excess stain was rinsed off by running under tap water. Next, the cells were air dried and the biofilm in the wells was documented microscopically (40×). Then, the crystal violet dye bound to biofilm in the wells was resolubilized with 160 μL 33% glacial acetic acid. The absorbance of each well was measured at 550 nm with a lid. The minimum biofilm inhibition concentration (MBIC) was the lowest concentration of an antimicrobial agent that resulted in no detectable biofilm growth [59], and was assessed visually. The half-maximal MBIC or median MBIC (MBIC_50_) was the concentration of an antimicrobial agent that led to 50% inhibition on the biofilm formation [60], and was calculated with the GraphPad Prism software with a mathematical model for a dose–response relationship (variable slope) after the normalization of the data and the logarithmic transformation of the applied concentrations (X-data).

### 2.9. Statistics

All experiments were performed in triplicate, including the positive, negative and untreated controls. For the cell redox and the biofilm assay, at least 8 wells were left for control and blank probes, and 2 to 4 wells for each concentration of the samples. One-way ANOVA was applied for the statistical analysis and a comparison of the groups of samples (GraphPad Prism software, Version 6.00, for Windows, GraphPad Software, La Jolla California, San Diego, CA, USA). The ANOVA data about result values different from the control are presented in the Supplementary Materials. A value of *p* < 0.05 was considered statistically significant.

## 3. Results

### 3.1. Extracts and Fractions: Phytochemical Composition of RochC, RochD and HirDM90 (the Most Active Samples)

**Figure 1:**
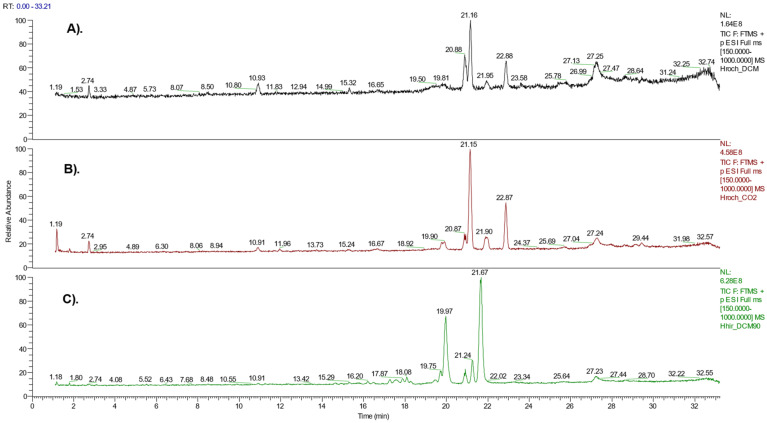
Total ion chromatogram (TIC) chromatographic profile of the samples with the highest antibacterial effect. The conditions and the time frame were the same for (**A**–**C**). (**A**) CH_2_Cl_2_ extract of aerial parts of *Hypericum rochelii* (RochD); (**B**) CO_2_ extract of aerial parts of *H. rochelii* (RochC); (**C**) fraction eluted with 90% MeOH on Diaion HP20 of CH_2_Cl_2_ extract of the aerial parts of *H. hirsutum* (HirDM90).

The phytochemical content was elucidated by means of the UHPLC–HRMS analysis (Figure 1), which revealed the following major secondary metabolites, all of them polyprenylated phloroglucinols (Table 1):

For the quantification of the main components, methoxyhyperibine J was selected as being a relatively more stable acylphloroglucinol derivative. The chromatographic conditions were optimized according to a procedure published elsewhere [63]. The contents of the constituents of the most active agents (Table 2) were calculated as methoxyhyperibine J using a quantifier ion at *m*/*z* 497.3625 with a 5 ppm isolation window. The calibration curve was built using polynomial regression which had a regression equation, correlation coefficient and concentration range of Y = 1 × 10^−15^ × X^2^ + 3 × 10^−6^ × X − 8.186, R^2^ = 0.9999 and 52.5–1680 ng/mL, respectively. For all compounds, except maculatoquiones A-D, the protonated molecules were selected as quantifier ions. For maculatoquiones A-D, the sum of the responses of the protonated molecule and fragment ion at *m*/*z* 345.2060 was due to the neutral loss of the isobutyric and 2-methylbutanoic acids. The total contents of the main constituents were 50.03%, 50.9% and 46.43% for RochC, RochD and HirDM90, respectively.

**Figure 2:**
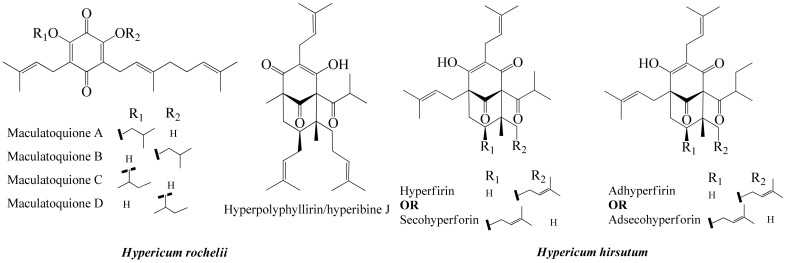
Phloroglucinols found in *H. rochelii* and *H. hirsutum*.

The origins of the samples and the phytochemical contents of the three most active ones are given in Table 3. The phloroglucinols found in them are given in Figure 2.

### 3.2. Antimicrobial Activity of the Extracts/Fractions

The results demonstrated that most of the samples had antibacterial activity, markedly more pronounced against Gram-positive bacteria, as expected, and three of them had a significant, and in some cases, an outstanding effect, comparable to the antibiotic used as a positive control (Table 4, Table 5, Table 6, Table 7 and Table 8) and comparable to the best results for MIC values of *Hypericum* extracts in the literature.

**Figure 3:**
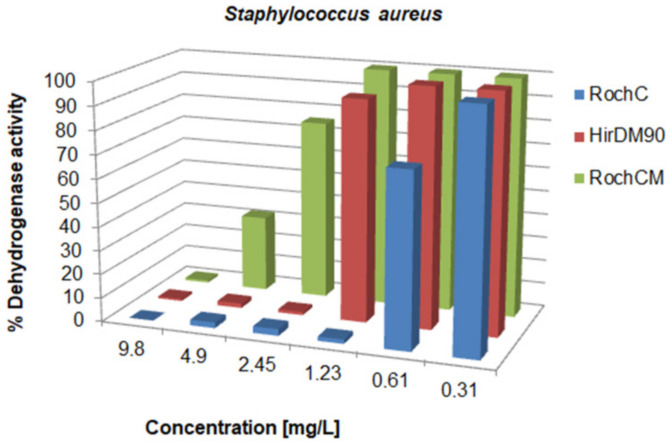
Metabolic activity of *S. aureus* after treatment with decreasing concentrations of three of the tested samples. The metabolic activity of untreated control was normalized as 100%. These samples, due to their high activity, were tested in a lower range of concentrations in order to obtain the antibacterial parameters.

**Figure 4:**
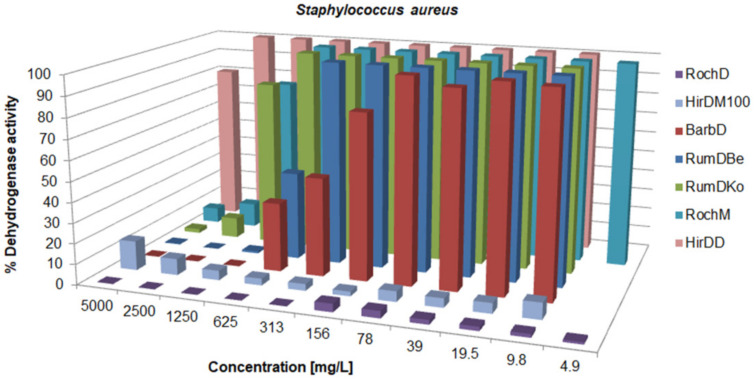
Metabolic activity of *S. aureus* after treatment with decreasing concentrations of seven of the tested samples. The metabolic activity of untreated control was normalized as 100%.

**Figure 5:**
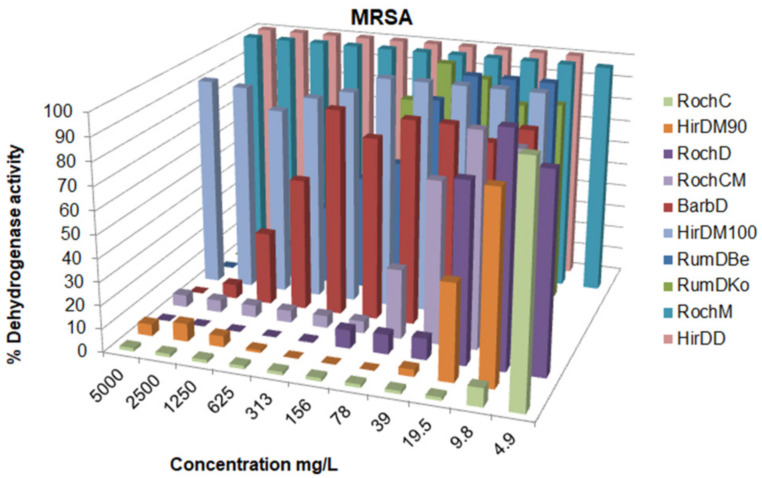
Metabolic activity of MRSA after treatment with decreasing concentrations of the tested samples. The metabolic activity of untreated control was normalized as 100%.

**Figure 6:**
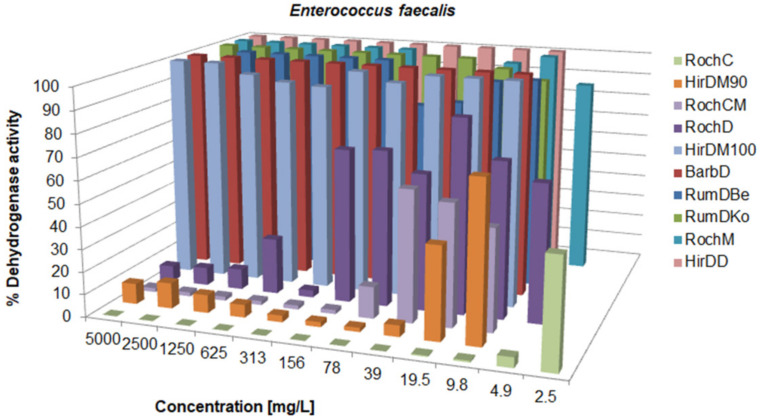
Metabolic activity of *E. faecalis* after treatment with decreasing concentrations of the tested samples. The metabolic activity of untreated control was normalized as 100%.

**Figure 7:**
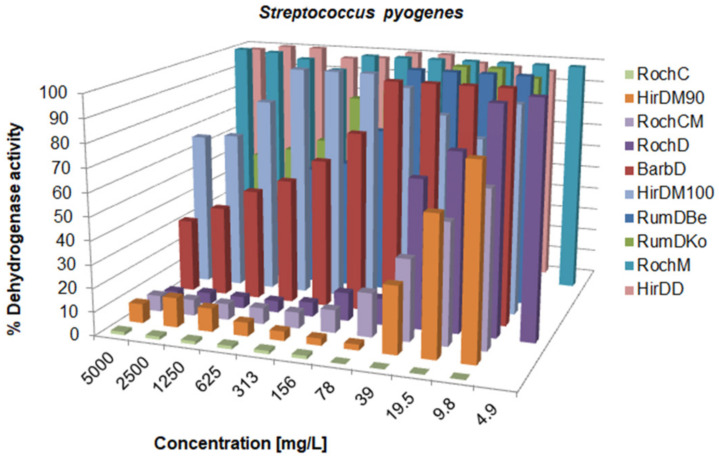
Metabolic activity of *S. pyogenes* after treatment with decreasing concentrations of the tested samples. The metabolic activity of untreated control was normalized as 100%.

**Figure 8:**
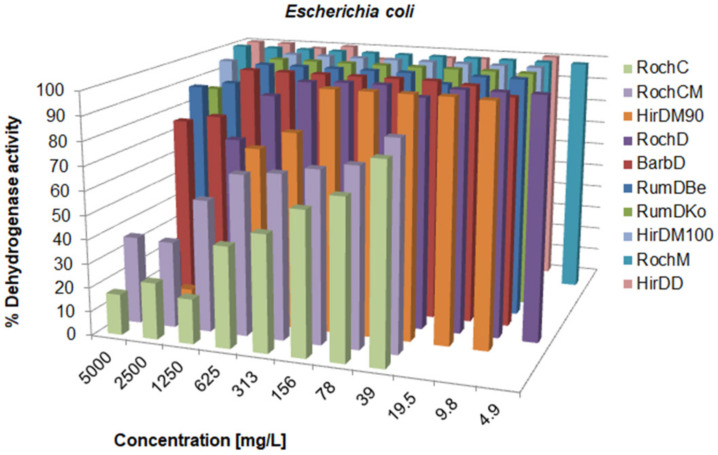
Metabolic activity of *E. coli* after treatment with decreasing concentrations of the tested samples. The metabolic activity of untreated control was normalized as 100%.

**Figure 9:**
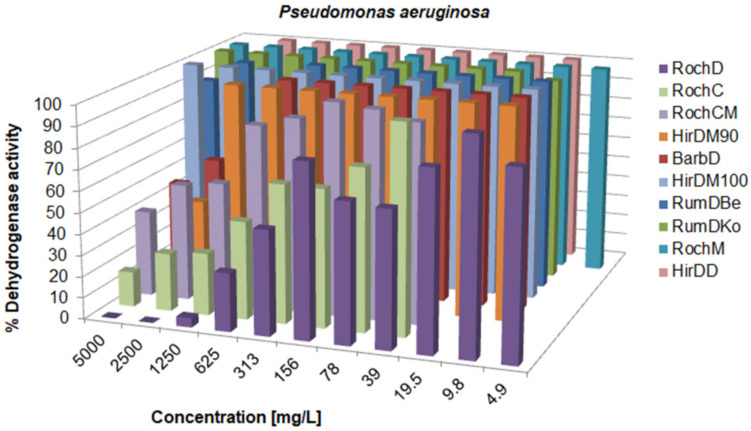
Metabolic activity of *P. aeruginosa* after treatment with decreasing concentrations of the tested samples. The metabolic activity of untreated control was normalized as 100%.

**Figure 10:**
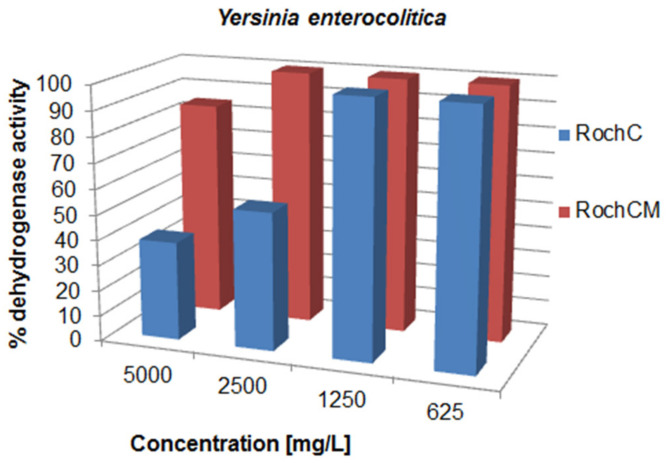
Metabolic activity of *Yersinia enterocolitica* after treatment with decreasing concentrations of the tested samples. The metabolic activity of untreated control was normalized as 100%.

There was a variation in the activity since the MIC values ranged from 0.625 to over 5000 mg/L; concentrations for the inhibition of CFU growth, determined on agar, ranged from 1.2 to >5000 mg/L; MBC values were in the interval from 19.5 to >5000 mg/L. Often, the MIC could not be determined because the extract was cloudy and resembled bacterial growth. If a certain extract had a MIC value over 5000 mg/L, it was not tested on agar, because such concentrations would be impractical in oral or topical drugs. The metabolic activity of the tested strains after treatment with a range of concentrations of the tested agents is given in Figure 3, Figure 4, Figure 5, Figure 6, Figure 7, Figure 8, Figure 9 and Figure 10. The metabolic activity of *C. albicans* was not inhibited using any extract; therefore, the graphs were not presented. As can be seen from Figure 3, Figure 4, Figure 5, Figure 6, Figure 7, Figure 8, Figure 9 and Figure 10, generally, as the MIC value was the lowest concentration that resulted in no visible bacterial growth, it was also the lowest concentration that resulted in a very low level of measured metabolic activity. The adjacent wells treated with a concentration lower than the MIC were marked with a rise in metabolic activity.

The most potent extract was, invariably, the one obtained with supercritical CO_2_ extraction (RochC). Its MIC, concentration for the inhibition of CFU growth and MBC against *S. aureus* were 0.625, 1.2 and 19.5 mg/L, respectively (Table 4). This MIC value was comparable to the positive control gentamicin and represented the highest antistaphylococcal activity of a crude *Hypericum* extract, in regard to MIC, to the best of our knowledge. Against the Gram-positive bacteria, this most effective extract had MICs of 0.625–19.5 mg/L, an inhibited CFU growth at 1.2–39 mg/L and MBCs were 19.5–156 mg/L.

The next most active samples were RochD and HirDM90, which generally had similar effects and also exerted their greatest influence on *S. aureus*. The MIC value of RochD was less or equal than the lowest concentration tested (≤4.9 mg/L), but we could attain the concentration for the inhibition of CFU growth (9.8 mg/L), and the MBC value was 78 mg/L. The last two parameters were higher than those of RochC, thus, the MIC for RochD had to be higher than 0.625 mg/L. The three parameters for HirDM90 were 2.5, 2.5 and 39 mg/mL. These findings were promising, since, as mentioned, *S. aureus* is one of the major human pathogens [3].

Actually, the extract RochCM, which was usually right behind HirDM90 in activity, also had an exceptional effect against the most susceptible strain, and with a MIC, an inhibition of CFU growth and MBC values of 4.9, 9.8 and 19.5 mg/L, respectively, was better than RochD.

RochD had MICs, an inhibition of CFU growth values and MBCs against Gram-positive bacteria of ≤4.9–78, 9.8–313 and 78–625 mg/L, respectively, and these parameters for HirDM90 were 2.5–39, 2.5–313 and 39–2500 mg/L, respectively.

As also mentioned, MRSA is becoming a common isolate of *S. aureus* [4], and though it was not as susceptible as the latter or *E. faecalis*, the MIC, inhibition of CFU growth and MBC values of the three best extracts in the range of 9.8–39, 9.8–313 and 78–625 mg/L, respectively, were also promising (Table 5). The results may not have been as good as those of *H. lanuginosum* Lam., which exerted a MIC value on MRSA of 0.78 mg/L [50], but were comparable to the other outstanding effect that we knew of, namely, the inhibitory concentration of 50 (IC_50_) < 5 mg/L for *H. laricifolium* Juss and other spp. [64].

Regarding *E. faecalis*, RochC and RochD outperformed the best results that we were aware of (MIC valued at 16 mg/L, exerted by *H. perforatum*) [65], but not the control antibiotics (Table 6).

The criteria of Eloff are used to this day, and they state that an extract or fraction has significant antibacterial activity if the MIC against the given organism is equal to or less than 100 μg/mL (or mg/L) [66]. As can be seen from Table 4, Table 5, Table 6 and Table 7, the three most active extracts, with a few exceptions, had MIC values much less than 100 mg/L against all Gram-positive strains, therefore, they had a very significant antibacterial activity. In some cases, especially with *S. aureus*, HrochCM and HirDM100 also fell within these criteria.

The least active agents were RochM and HirDD with MIC values of >5000 mg/L, but even they, especially RochM, inhibited the metabolic activity of *S. aureus* at 2500 and 5000 mg/L (Figure 4). HirDD is rich in lipids, but probably does not contain many long-chain free polyunsaturated fatty acids, which are known to be antibacterial [67]. The other extracts had intermediate effects. The extract HirDM100 had the greatest difference between the MIC values and those for the inhibition of the CFU growth, meaning it was bacteriostatic but not bactericidal.

Most often, there was a prominent difference between the MIC values and the concentrations for the inhibition of the CFU growth, and between the latter and the MBC, which meant that the extracts were bacteriostatic in a broad range of concentrations.

In regard to the Gram-negative bacteria and *C. albicans*, generally, the samples were inactive up to 5000 mg/L. However, there was an exception we considered important concerning *Y. enterocolitica*—RochC had a concentration for the inhibition of the CFU growth of 5000 mg/mL, and a respective decrease in the metabolic activity at 5000 and 2500 mg/L was present. Additionally, the strongest agents demonstrated an inhibition of the metabolic activities of *E. coli* and *P. aeruginosa* in the highest concentrations tested (625–5000 mg/L).

### 3.3. Antibiofilm Activity of the Tested Active Ingredients

**Figure 11:**
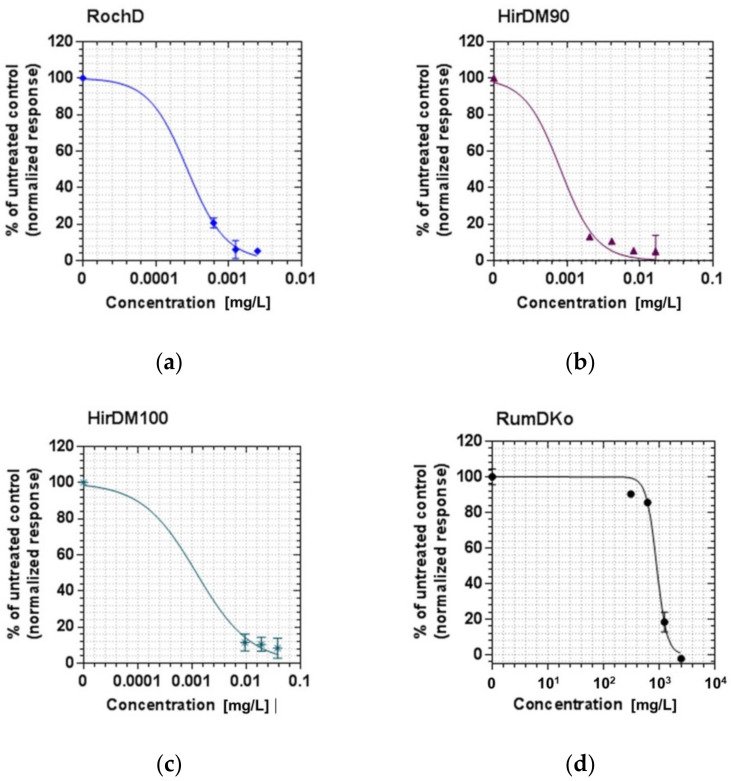
Biofilm inhibition of MRSA after exposure to four samples. (**a**) Sample RochD; (**b**) Sample HirDM90; (**c**) Sample HirDM100; (**d**) Sample RumDKo.

**Figure 12:**
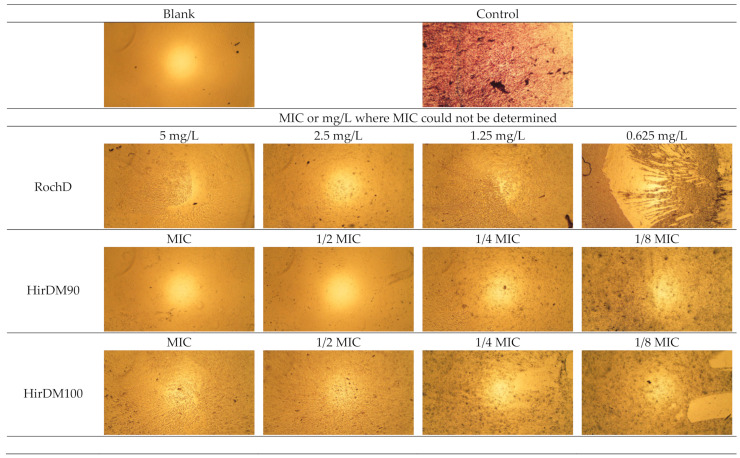
Microscopic evaluation of the biofilm inhibition using four samples—40× magnification.

The samples we tested (RochD, HirDM90, HirDM100 and RumDKo) had a concentration-dependent biofilm-inhibitory activity against MRSA (Figure 11 and Figure 12 and Table 9), which corresponded to their overall activity. Their MBIC and MBIC_50_ values were lower than their MBC values, as well as their MIC values, except for RumDKo. The MBIC and MBIC_50_ of RochD were only 2.5 mg/L and 0.27 mg/L, respectively. These parameters were better than the lowest reported MBIC_50_ value for MRSA that we were able to find (approximately 8 mg/L belonging to *H. lydium* Boiss.) [68], and were close to the values of single compounds [59,69].

## 4. Discussion

Our data showed that the lipophilic extract of a species, i.e., obtained with a nonpolar (CH_2_Cl_2_, CO_2_) solvent, resulted in a more active extract than its counterpart obtained with a more polar (MeOH) solvent or a mix between the two. Though this finding was valid to other reports too [44,70], we could not state it is a rule, since in many cases, it was an alcohol extract that held a high potency [46,49,71]. The supercritical CO_2_ extraction turned out to be most effective in our study, in terms of producing antibacterial extracts, but this process took a long time to exhaust the plant material, hence, it was less profitable from an economic point of view.

The phloroglucinol content in RochC and RochD turned out to be the same, and this probably determined their similar activity, being the greatest in this report. The prevalent compounds in them were found to be PPAPs, or, more precisely, bicyclic polyprenylated acylphloroglucinols (BPAPs) with a bicyclo[3.3.1]nonane-2,4,9-trione skeleton of hyperforin-type, or, in other words, hyperforins. To the best of our knowledge, none of the individual compounds found in this work were tested for antimicrobial activity. However, since hyperforins have superior antibacterial effects among phloroglucinols (e.g., pure hyperforin had a MIC of 0.1 mg/L against *S. aureus* and *Sarcina lutea*) [22], it was safe to assume that the extracts and fractions in the present work owed their activity to hyperforins.

Regarding previous research on the chemical profile of the four species, we found there was one report about *H. barbatum*; *H. rochelii* was examined little, including one very recent study; *H. hirsutum* was studied quite extensively.

The main phenolic compounds found in *H. barbatum* were hyperoside, 5-*O*-caffeoylquinic acid and quercitrin; interestingly, there was a higher hypericin content than in *H. perforatum* [72].

*H. rochelii* is a Balkan species that grows at altitudes of 500–1200 m on calcareous rocks [73]. Specimens have been found to contain phenolic acids, myricetin rhamnosides, other flavonoids in large amounts [74] and also the glucosylxanthones isomangiferin and mangiferin [75]. The authors focused on the polar phenolic compounds and did not comment on the presence of phloroglucinols. It is possible that they had transformed before the extraction.

*H. hirsutum*, commonly known as hairy St John’s wort, grows in Europe and western Siberia in open or partially shaded habitats [76]. By far, the groups of phloroglucinols and flavonoids in the plant are the most numerous. Its hypericin [77] and hyperforin [78] contents are lower than in *H. perforatum* [72,79]. The plant also contains many PPAPs with homoadamantane and adamantane skeletons [80], phenolic acids [81] and other phenolic compounds [82], such as avicularin [78], amentoflavone [83], etc.

The MICs of *Hypericum* extracts are usually lower than 1000 mg/L, and often less than 100 mg/L, thus, fulfilling the criteria of Eloff. To the best of our knowledge, *H. perforatum* produced the most active extract with a MIC of 0.625 mg/L against *Lactobacillus* spp. [50]. The next best results belonged to *H. lanuginosum*, with a MIC of 0.78 mg/L against *S. aureus*, MRSA and the fungi *C. albicans*, with a MIC 0.78 mg/L and comparable or more potent activity than the control antibiotic [50]. Therefore, a good antifungal effect of St John’s wort spp. may be rare, but could still be found. The results were even more surprising because the extract was aqueous, and water extracts, as a rule, are the least active *Hypericum* extracts [28,48]. While the effect of the *H. perforatum* extract could be attributed to the high amount of hypericin (0.1–0.2 mg/mL) [84], the aqueous extract could not contain prenylated phloroglucinols, but had the highest amount of phenols, flavonoids and tannins. It was not commented on whether the phenols contained hypericins [50].

These two reports were extraordinary, because the MICs of the crude extracts were close to the MICs of single compounds, with St John’s wort values ranging at 0.8–16 μM [85]. For example, as mentioned, pure hyperforin had a MIC of 0.1 mg/L against *S. aureus* and *Sarcina lutea*, and, in addition, 1 mg/L against MRSA, *E. faecalis* and other Gram-positive bacteria, as well as 400 mg/L against Gram-negative ones, including *E. coli* and fungi, including *C. albicans* [22]. This fact illustrated well the typical stronger effect of some St John’s wort components on Gram-positive bacteria in comparison to Gram-negative ones and fungi. The preparation novoimanine also had a MIC value of 0.1 mg/L on *S. aureus*, and was more effective than sulfanilamide [25]. In contrast, the lowest MIC of hypericin was 0.18 mg/L against pathogenic fungi and spoilage yeasts, but, in another study, it could reach 12–47 mg/L against other bacteria and fungi [23,86].

Additionally, as expected, *Hypericum* extracts were quite potent against *S. aureus*, with MICs on a multidrug-resistant (MDR) strain of 4 mg/L for *H. brasiliense* [87]. In some cases though, the MeOH extract from *H. perforatum* could give MICs against *S. aureus* and staphylococci isolated from cow mastitis as high as 813 mg/L [88]. The best activity against Gram-negative bacteria that we know of was the MIC of 7.8 mg/L against *Helicobacter pylori* that belonged to a fraction from the MeOH extract of *H. perforatum* [71]. Only the ethyl acetate fraction from the methanol extract from the bark of *H. roeperianum* Schimp. ex A.-rich shrub had MICs of 16 and 32 mg/L against *E. coli* and *P. aeruginosa*, respectively, even MDR ones [46].

The methanol extracts of several Balkan St John’s wort spp. were tested by Radulovic et al. [45]; among them, the objects of this study, *H. barbatum*, *H. rumeliacum* and *H. hirsutum*, demonstrated good antibacterial activity. *H. hirsutum* was particularly active, with inhibition zones in some cases reaching more than twice the value of the antibiotics used as positive controls, and, interestingly, against both Gram-negative bacteria such as *E. coli*, Gram-positive bacteria (and *S. aureus* was not nearly as susceptible as *S. enteritidis*) and fungi such as *Aspergillus niger*. Significant values of inhibition were also obtained from methanol–acetone extracts from *H. hirsutum* against *S. aureus* and *P. aeruginosa*, but not as high as those of *H. perforatum*, and not against *E. faecalis*, *E. coli* and *C. albicans* [79].

However, it was difficult to compare those antimicrobial results with ours because of the different methodology—they used the disc diffusion and the agar well diffusion methods.

A recently published paper, which also used the microdilution method, showed that a 70% ethanol extract of *H. rochelii* from Romania had a significantly lower activity for Gram-positive bacteria, especially *S. aureus*, than our CO_2_ and CH_2_Cl_2_ extracts. Its MIC and MBC values were 250–1000 and 500–2000 mg/L, respectively [74]. It is possible that this was due to the polyprenylated phloroglucinols found in the extracts in the present study. Yet, the extract obtained by Babotă et al. had an effect on Gram-negative bacteria (e.g., *E. coli* and *Salmonella typhymurium*), which was not substantially different from the effect on Gram-positive ones. Tested on fungi such as *Aspergillus* and *Penicillium* and not *C. albicans*, some of the MICs were 2000 mg/L, and some of the MBCs were 4000 mg/L, rendering this specimen a more active fungicide than ours. Interestingly, *H. perforatum* had a similar activity profile.

The essential oil of all four species in this work was also tested for antibacterial effects using the broth microdilution assay, and their effects varied from weak to significant [89,90,91,92]. However, essential oils mainly contain volatile compounds, and were not very relevant to this study. Moreover, *Hypericum* spp. were classified as essential-oil-poor plants, usually yielding less than 1% *w*/*w* [93].

Regarding the antibiofilm effect, there are few reports about the *Hypericum* active ingredients and MRSA or *S. aureus* biofilms. An ethanol extract from *H. lydium* had a MBIC_50_ value on clinical isolates of MRSA and *S aureus* of approximately 8 mg/L [68]. Again, an ethanol extract, but from *H. perforatum*, had a MBIC_50_ for the MRSA biofilm of 128 mg/L, lower than the inhibitory concentration for planktonic growth [94]. An extract from *H. brasiliense* failed to show MBIC or MBIC_50_ values equal or lower than its very low MIC value of 4 mg/L on the MDR *S. aureus* strain for a mature biofilm grown for 24 h, only for a forming and six-hour-old biofilm [87]. A polyurethane material combined with *H. perforatum* extract inhibited the formation of *S. aureus* biofilm [95]. Traditional oil macerates from the same species inhibited biofilm formation from an *S. aureus* biofilm test strain to some degree in the absence of any inhibition on its planktonic growth. A MeOH extract had both MBIC and MIC values of 16 mg/L, a commercial liquid supplement had a MBIC value of 128 mg/L (about 1/2 MIC) and an aqueous decoction (all from aerial parts) had a MBIC_50_ value of 512 mg/L, in contrast to its MIC of 64 mg/L [96]. As to individual compounds, five bioactive phloroglucinol derivatives from four *Hypericum* species had MBIC against biofilm from MRSA 3.91–7.81 mg/L, but the MBIC value was lower than its MIC and MBC values for only one of them. Their MBICs against the biofilms from *S. aureus* and *Staphylococcus epidermidis* were 1.95–7.81 mg/L, and most of these values were lower than the respective MICs and MBCs [59]. A dicyclohexylamonium salt of hyperforin and its hydrogenated analogue had MBICs against the biofilm of a MRSA clinical isolate at 25–37.5 mg/L, and on the biofilm of *S. aureus* and *E. faecalis*, their MBICs were 25 to 150 mg/L, which were much higher than their MICs of 1–4 mg/L [69]. To give an example with another bacterial species, *H. perforatum* extracts had a MBIC_50_ of approximately 7–8 mg/L against *Streptococcus sobrinus*, the most susceptible strain in the study, also found in dental plaque [65].

When we consider candidates for drugs or food additives, bioavailability and interactions with the (gut) microbiota are important factors. Concerning the bioavailability, there are data about the poor pharmacokinetic profile of St John’s wort extracts, i.e., low bioavailability (15–20%), which is mainly due to the very poor water solubility of the active molecules, such as hyperforin, hypericin and rutin. This may be the cause of the 4–6-week treatment period required to achieve a therapeutic benefit in patients with depression. Still, antidepressants with better pharmacokinetic profiles also typically require a similar treatment period before therapeutic effects are seen [97,98]. Wurglics and Schubert-Zsilavecz [99] summarized the bioavailability data. Hyperforin seems to be the only St John’s wort component capable of crossing the blood–brain barrier. Its plasma concentration in humans reached approximately 300 ng/mL after an oral administration of a 600 mg extract (containing 5% hyperforin). This was very close to the therapeutic antidepressant concentrations of hyperforin. Higher doses and repeated once-daily doses of the extract led to less bioavailability, mainly due to the high lipophilicity of hyperforin and other factors. Compared with hyperforin, the plasma concentrations of the hypericins were only one-tenth, and of caffeic acid [100] and flavonoids approximately a half, despite the generally poor absorption of flavonoids that may result from poor solubility and other factors [101]. It was found that flavonol glycosides were not absorbed intact after an oral dose, but significant plasma concentrations of the aglycones were detected. Additionally, the bioavailability of an aglycone after the ingestion of its diglycosides was approximately half of that after the intake of its monoglucosides. It is interesting that flavonoids, especially hyperoside and procyanidins, increase the water solubility of hypericins up to 400-fold, which can lead to better bioavailability [102]. Other prenylated phloroglucinols from *Hypericum* or phloroglucinol derivatives from other plants also have limited oral bioavailability [103,104] because of significant lipophilicity and low water solubility, predicted with in silico studies. Nanonization, liposomal preparations and synthetic strategies that decrease their lipophilicity, simplify their structure and eliminate troublesome functionalities can make them suitable for oral drug leads [98,105].

In regard to the microbiota interactions, it is known that hydrolyzed and/or fission products are derived from herbal polyphenols through intestinal bacteria. These catabolites exert their physiological functions in target sites after transportation and/or could affect the microbiome in place, resulting in health promotion, e.g., through the intestinal immune function. Flavonoids and oligomeric proanthocyanidins are usually catabolized to chain fission products by intestinal bacteria in the colon [106]. However, there is no research specifically about the similar fate of PPAPs or other compounds from *Hypericum*. Still, the fate of some flavonoids could be unraveled, since we know, for example, that the maximum plasma peak of the diglycoside rutin was significantly delayed (7 h versus 0.7 h for the monoglucosides), which indicated not an absorption in the small intestine, but in the terminal ileum after microbial degradation [99]. In contrast, it is known that one of the products of some phenolic substances, e.g., catechins and various flavonoids, produced by mammal gut microbiota is simple phloroglucinol [107,108,109], and the latter is, finally, turned into volatile fatty acids by rumen microbiota [110].

The influence of *Hypericum* ingredients on the oral *Lactobacillus* microflora, which are the main participants in oral infections and dental caries in the first years of our lives, has been studied, albeit poorly. *Hypericum* ingredients have antibacterial effects against the described lactobacilli [65,111,112,113] and could be developed into oral care products.

The current research is the first work to assess the antimicrobial potential of the four *Hypericum* species from the Bulgarian population and the first report on the phloroglocunols found in *H. rochelii*.

## 5. Conclusions

The four *Hypericum* species included in the presented study exhibited from weak to extraordinary antibacterial activity on a panel of pathogenic Gram-positive and Gram-negative bacterial strains, with some samples from them having exceptionally high antibiofilm activity against MRSA. The highest potential to inhibit bacterial growth and biofilm inhibition was observed for *H. rochelii* and *H. hirsutum.* The former presented the best antistaphylococcal results for the genus in regard to the MIC, that we know of, comparable to the activity of gentamicin and pure antibacterial phytochemicals from St John’s wort. The evaluation of the phytochemical content of the extracts revealed their potential as rich sources of biologically active polyprenylated phloroglucinols. The data obtained not only contribute to the better pharmacological characterization of the tested extracts and fractions, but are promising in terms of further development in the most potent active ingredients as food additive or drug candidates with antibacterial effects for the eradication of pathogenic bacteria, which is a very important perspective in light of increasing antimicrobial resistance. Other future works could include more fractionation and isolation of single compounds from the samples, as well as the optimization of the supercritical CO_2_ extraction to make it more feasible from an economic point of view.

## Figures and Tables

**Table 1:** Tentative identification of main components of the most active samples.

Rt[min] ^1^	Compound	[M+H]^+^	∆ppm	Molecular Formula	MS/MS (Intensity)	Sample	Lit.
19.49	Hyperfirin or Secohyperforin	469.3313	0.14	C_30_H_45_O_4_	413.27 (14), 401.27 (14), 345.21 (100), 343.23 (57), 277.14 (40)	HirDM90 (Figure 1C)	[61,62]
19.77	Maculatoquione A ^2^	415.2477	−0.60	C_25_H_35_O_5_	345.21 (100), 291.12 (13), 289.14 (14), 223.10 (21), 221.08 (22)	RochD, RochC (Figure 1A,B)	[53]
19.90	Maculatoquione B ^2^	415.2478	−0.24	C_25_H_35_O_5_	345.21 (100), 289.14 (16), 223.10 (22), 221.08 (15)	RochD, RochC (Figure 1A,B)	[53]
19.97	Hyperfirin or Secohyperforin	469.3313	0.07	C_30_H_45_O_4_	413.27 (18), 401.28 (52), 345.21 (61), 343.23 (67), 333.21 (11), 277.14 (100), 275.14 (11), 137.13 (11)	HirDM90 (Figure 1C)	[61,62]
21.15	Hyperpolyphyllirin/Hyperibine J ^2^	483.3462	−1.38	C_31_H_47_O_4_	427.28 (14), 279.16 (84), 223.10 (100), 205.20 (57), 149.13 (67), 135.12 (29), 95.09 (61)	RochD, RochC (Figure 1A,B)	[53]
21.26	Adhyperfirin or Adsecohyperforin	483.3470	0.19	C_31_H_47_O_4_	427.28 (17), 415.28 (17), 359.22 (100), 343.23 (93), 291.10 (43)	HirDM90 (Figure 1C)	[61,62]
21.67	Adhyperfirin or Adsecohyperforin	483.3469	0.01	C_31_H_47_O_4_	427.28 (20), 415.28 (45), 359.22 (56), 343.23 (100), 291.16 (87), 275.16 (14)	HirDM90 (Figure 1C)	[61,62]
21.88	Maculatoquione C ^2^	429.2636	0.07	C_26_H_37_O_5_	345.21 (100), 289.14 (15), 223.10 (20), 221.08 (24)	RochD, RochC (Figure 1A,B)	[53]
21.98	Maculatoquione D ^2^	429.2635	−0.07	C_26_H_37_O_5_	345.21 (100), 289.14 (14), 223.10 (20), 221.08 (16)	RochD, RochC (Figure 1A,B)	[53]
22.87	Unknown	497.3625	−0.02	C_32_H_49_O_4_	441.30 (14), 293.17 (71), 237.11 (100), 205.20 (56), 149.13 (61), 135.12 (24), 95.09 (58)	RochD, RochC (Figure 1A,B)	[53]

^1^ The retention times were taken from XIC chromatograms. See supporting materials. ^2^ Compounds were identified through comparison with authentic samples.

**Table 2:** Contents of main components of the most active samples.

Rt (min)	Compound	Quantifier Ion	% Contents ± SD		
			RochC	RochD	HirDM90
19.49	Hyperfirin or Secohyperforin	469.3312	N.D. ^1^	N.D. ^1^	0.37 ± 0.01
19.76–19.88	Maculatoquione A and Maculatoquione B ^2^	415.2477 and 345.2060	1.62 ± 0.04	1.48 ± 0.02	N.D. ^1^
19.97	Hyperfirin or Secohyperforin	469.3312	N.D. ^1^	N.D. ^1^	15.19 ± 0.09
21.15	Hyperpolyphyllirin/Hyperibine J	483.3469	30.50 ± 0.69	31.11 ± 0.50	N.D. ^1^
21.26	Adhyperfirin or Secoadhyperforin	483.3469	N.D. ^1^	N.D. ^1^	2.73 ± 0.08
21.67	Adhyperfirin or Secoadhyperforin	483.3469	N.D. ^1^	N.D. ^1^	28.14 ± 0.42
21.88–21.98	Maculatoquione C and Maculatoquione D ^2^	429.2636 and 345.2060	2.77 ± 0.06	2.66 ± 0.03	N.D. ^1^
22.87	Unknown	497.3625	15.14 ± 0.32	15.65 ± 0.20	N.D. ^1^

^1^ Not detected. ^2^ Isobaric compounds were not possible to quantify separately due to poor chromatographic separation.

**Table 3:** Extracts and fractions.

Sample	Species	Extraction and Extractant	Phytochemicals Known to Be Present or Found
RochC	*Hypericum rochelii*	Supercritical CO_2_ extraction ^1^–CO_2_	Maculatoquiones A, B, C and D, hyperpolyphyllirin/hyperibine J and unknown
RochD	*H. rochelii*	Dichloromethane (CH_2_Cl_2_)	Maculatoquiones A, B, C and D, hyperpolyphyllirin/hyperibine J and unknown
HirDM90	*H. hirsutum*	90% aq. methanol (MeOH) fraction from CH_2_Cl_2_ extract ^2^	Hyperfirin or secohyperforin, adhyperfirin or adsecohyperforin
RochCM	*H. rochelii*	Supercritical CO_2_ extraction–CO_2_ with modifier MeOH	
HirDM100	*H. hirsutum*	MeOH fraction from CH_2_Cl_2_ extract	
BarbD	*H. barbatum*	CH_2_Cl_2_	
RumDBe	*H. rumeliacum* (Bela voda, Pernik)	CH_2_Cl_2_	
RumDKo	*H. rumeliacum* (Konyavska mountain)	CH_2_Cl_2_	
RochM	*H. rochelii*	MeOH and aq. MeOH (after the plant material was extracted with CH_2_Cl_2_)	
HirDD	*H. hirsutum*	CH_2_Cl_2_ fraction from CH_2_Cl_2_ extract	Lipids, chlorophyll and waxes

^1^ If not specified, all other samples were a result of conventional extraction. ^2^ If not specified, all other samples were extracts.

**Table 4:** Antimicrobial activity of the tested extracts and fractions on *Staphylococcus aureus*.

Bacterial Strain	*Staphylococcus aureus*		
Parameters	Minimal Inhibitory Concentration (MIC)	Inhibition of Colony-Forming Unit (CFU) Growth	Minimum Bactericidal Concentration (MBC)
Extract or Fraction (mg/L)			
RochC	0.625	1.2	19.5
RochD	≤4.9	9.8	78
HirDM90	2.5	2.5	39
RochCM	9.8	4.9	19.5
HirDM100	≤9.8	156	2500
BarbD	- ^1^	1250	2500
RumDBe	-	5000	-
RumDKo	-	>5000	-
RochM	-	>5000	-
HirDD	-	>5000	-
**Referent Antibiotic (mg/L)**			
Gentamicin	0.25	-	-

^1^ Cannot be determined, e.g., because the extract was cloudy and resembled bacterial growth.

**Table 5:** Antimicrobial activity of the tested extracts and fractions on MRSA.

Bacterial Strain	Methicillin-Resistant *S. aureus*		
Parameters	MIC	Inhibition of CFU Growth	MBC
Extracts or Fractions (mg/L)			
RochC	9.8	9.8	78
RochD	-	39	313
HirDM90	39	313	625
RochCM	156	313	2500
HirDM100	156	>5000	-
BarbD	-	5000	-
RumDBe	-	5000	-
RumDKo	2500	5000	-
RochM	-	>5000	-
HirDD	-	>5000	-
**Referent Antibiotic (mg/L)**			
Gentamicin	0.125		

**Table 6:** Antimicrobial activity of the tested extracts and fractions on *Enterococcus faecalis*.

Bacterial Strain	*Enterococcus faecalis*		
Parameters	MIC	Inhibition of CFU Growth	MBC
Extracts or Fractions (mg/L)			
RochC	4.9	9.8	78
RochD	≤4.9	39	156
HirDM90	39	156	625
RochCM	156	156	1250
HirDM100	78	>5000	-
BarbD	5000	>5000	-
RumDBe	5000	>5000	-
RumDKo	5000	>5000	-
RochM	-	>5000	-
HirDD	5000	>5000	-
**Referent Antibiotics (mg/L)**			
Penicillin	2.5		
Gentamicin	8		
Ciprofloxacin	0.5		

**Table 7:** Antimicrobial activity of the tested extracts and fractions on *Streptococcus pyogenes*.

Bacterial Strain	*Streptococcus pyogenes*		
Parameters	MIC	Inhibition of CFU Growth	MBC
Extracts or Fractions (mg/mL)			
RochC	19.5	39	156
RochD	78	313	625
HirDM90	39	156	2500
RochCM	-	2500	5000
HirDM100	625	>5000	-
BarbD	-	>5000	-
RumDBe	2500	>5000	-
RumDKo	5000	>5000	-
RochM	2500	>5000	-
HirDD	5000	>5000	-
**Referent Antibiotic (mg/L)**			
Penicillin	0.08		

**Table 8:** Antimicrobial activity of the tested extracts and fractions on *Escherichia coli*, *Pseudomonas aeruginosa*, *Yersinia enterocolitica* and *Candida albicans*.

Bacterial Strain	*Escherichia coli*, *Pseudomonas aeruginosa*, *Yersinia enterocolitica* and *Candida albicans*		
Parameters	MIC	Inhibition of CFU Growth	MBC
Extracts or Fractions (mg/L)			
RochC ^1^	-	-	-
RochD	-	>5000	-
HirDM90	-	>5000	-
RochCM	-	-	-
HirDM100	-	>5000	-
BarbD	>5000	-	-
RumDBe	>5000	-	-
RumDKo	>5000	-	-
RochM	-	>5000	-
HirDD	-	>5000	-
**Referent Antibiotics/Chemotherapeutics (mg/L)**			
For *E. coli*: gentamicin	2	-	-
For *P. aeruginosa*: gentamicin	0.5	-	-
For *Y. enterocolitica*: ciprofloxacin	0.0125	-	-
For *C. albicans*: amphotericin B	0.125	-	-

^1^ This extract was an exception, and was the only one with a MIC on *Yersinia enterocolitica*, 5 mg/mL.

**Table 9:** Biofilm inhibition activity of four extracts on MRSA (in mg/L).

Extract	Minimum Biofilm Inhibitory Concentration (MBIC)	Median Biofilm Inhibitory Concentration (MBIC_50_)
RochD	2.5	0.27 (0.18 to 0.42)
HirDM90	19.5 (1/2 MIC)	0.82 (0.51 to 1.32)
HirDM100	39 (1/4 MIC)	1.21 (0.40 to 3.63)
RumDKo	2500	910 (825 to 999)

## Data Availability

All raw data from the experiments are available from the authors.

## Supplementary Materials

The following supporting information can be downloaded at: https://www.mdpi.com/article/10.3390/life13020274/s1, Figure S1: chromatographic profile of RochD; Figure S2: chromatographic profile of RochC; Figure S3: chromatographic profile of HirDM90; Table S1: one-way ANOVA of the metabolic activity of the tested bacteria. Column statistics; Table S2: one-way ANOVA of the metabolic activity of *Staphylococcus aureus*. Comparison between the treated groups and untreated control; Table S3: one-way ANOVA of the metabolic activity of MRSA. Comparison between the treated groups and untreated control; Table S4: one-way ANOVA of the metabolic activity of *Enterococcus faecalis*. Comparison between the treated groups and untreated control. Table S5: one-way ANOVA of the metabolic activity of *Streptococcus pyogenes*. Comparison between the treated groups and untreated control; Table S6: one-way ANOVA of the metabolic activity of *Eschericia coli*. Comparison between the treated groups and untreated control; Table S7: one-way ANOVA of the metabolic activity of *Pseudomonas aeruginosa*. Comparison between the treated groups and untreated control; Table S8: one-way ANOVA of the metabolic activity of *Yersinia enterocolitica*. Comparison between the treated groups and untreated control; Table S9: one-way ANOVA of the biofilm absorbance. Column statistics; Table S10: one-way ANOVA of the biofilm absorbance. Comparison between the treated groups and untreated control.
