# Supplementary material for: Outstanding Antibacterial Activity of Hypericum rochelii—Comparison of the Antimicrobial Effects of Extracts and Fractions from Four Hypericum Species Growing in Bulgaria with a Focus on Prenylated Phloroglucinols
Source: Life (Basel). 2023 Jan 18;13(2):274. doi: 10.3390/life13020274 (PMC9959064; doi:10.3390/life13020274)
Supplement: Supplementary file 1 [file life-13-00274-s001.zip › life-1975708-supplementary/Suppl. Table S2 DEHA S. aureus statistics.pdf]

Table S2. One-way ANOVA of the metabolic activity of *Staphylococcus aureus*. Comparison between the treated groups and untreated control.

Extract	Dunnett's multiple comparisons test	Significance		Adjusted P Value
RochC	Untreated control vs. 1250*	Yes	****	< 0,0001
	Untreated control vs. 625	Yes	****	< 0,0001
	Untreated control vs. 313	Yes	****	< 0,0001
	Untreated control vs. 156	Yes	****	< 0,0001
	Untreated control vs. 78	Yes	****	< 0,0001
	Untreated control vs. 39	Yes	****	< 0,0001
	Untreated control vs. 19,5	Yes	****	< 0,0001
	Untreated control vs. 9,8	Yes	****	< 0,0001
	Untreated control vs. 4,9	Yes	****	< 0,0001
	Untreated control vs. 2,5	Yes	****	< 0,0001
	Untreated control vs. 1,23	Yes	****	< 0,0001
	Untreated control vs. 0,61	Yes	**	0,0045
HirDM90	Untreated control vs. 5000	Yes	****	< 0,0001
	Untreated control vs. 2500	Yes	****	< 0,0001
	Untreated control vs. 1250	Yes	****	< 0,0001
	Untreated control vs. 625	Yes	****	< 0,0001
	Untreated control vs. 313	Yes	****	< 0,0001
	Untreated control vs. 156	Yes	****	< 0,0001
	Untreated control vs. 78,125	Yes	****	< 0,0001
	Untreated control vs. 39,0625	Yes	****	< 0,0001
	Untreated control vs. 19,5	Yes	****	< 0,0001
	Untreated control vs. 9,8	Yes	****	< 0,0001
	Untreated control vs. 4,9	Yes	*	0,0136
	Untreated control vs. 2,5	Yes	*	0,0142
	Untreated control vs. 1,23	Yes	*	0,0139
	Untreated control vs. 0,61	No	ns	0,9946
RochD	Untreated control vs. 5000	Yes	****	< 0,0001
	Untreated control vs. 2500	Yes	****	< 0,0001
	Untreated control vs. 1250	Yes	****	< 0,0001
	Untreated control vs. 625	Yes	****	< 0,0001
	Untreated control vs. 313	Yes	****	< 0,0001
	Untreated control vs. 156	Yes	****	< 0,0001
	Untreated control vs. 78	Yes	****	< 0,0001
	Untreated control vs. 39	Yes	****	< 0,0001
	Untreated control vs. 19,5	Yes	****	< 0,0001
	Untreated control vs. 9,8	Yes	****	< 0,0001
	Untreated control vs. 4,9	Yes	****	< 0,0001
RochCM	Untreated control vs. 5000	Yes	****	< 0,0001
	Untreated control vs. 2500	Yes	****	< 0,0001
	Untreated control vs. 1250	Yes	****	< 0,0001
	Untreated control vs. 625	Yes	****	< 0,0001
	Untreated control vs. 313	Yes	****	< 0,0001
	Untreated control vs. 156	Yes	****	< 0,0001
	Untreated control vs. 78,125	Yes	****	< 0,0001
	Untreated control vs. 39,0625	Yes	****	< 0,0001
	Untreated control vs. 19,5	Yes	****	< 0,0001
	Untreated control vs. 9,8	Yes	****	< 0,0001
	Untreated control vs. 4,9	Yes	**	0,0061
	Untreated control vs. 2,5	Yes	*	0,0296
BarbD	Untreated control vs. 5000	Yes	****	< 0,0001
	Untreated control vs. 2500	Yes	****	< 0,0001
	Untreated control vs. 1250	Yes	****	< 0,0001
	Untreated control vs. 625	Yes	****	< 0,0001
	Untreated control vs. 313	Yes	****	< 0,0001
	Untreated control vs. 156	No	ns	0,0625
HirDM100	Untreated control vs. 5000	Yes	****	< 0,0001
	Untreated control vs. 2500	Yes	****	< 0,0001

	Untreated control vs. 1250	Yes	****	< 0,0001
	Untreated control vs. 625	Yes	****	< 0,0001
	Untreated control vs. 313	Yes	****	< 0,0001
	Untreated control vs. 156	Yes	****	< 0,0001
	Untreated control vs. 78,125	Yes	****	< 0,0001
	Untreated control vs. 39,0625	Yes	****	< 0,0001
	Untreated control vs. 19,5	Yes	****	< 0,0001
	Untreated control vs. 9,8	Yes	****	< 0,0001
RumDBe	Untreated control vs. 5000	Yes	****	< 0,0001
	Untreated control vs. 2500	Yes	****	< 0,0001
	Untreated control vs. 1250	Yes	****	< 0,0001
	Untreated control vs. 625	Yes	****	< 0,0001
RumDKo	Untreated control vs. 5000	Yes	****	< 0,0001
	Untreated control vs. 2500	Yes	****	< 0,0001
	Untreated control vs. 1250	No	ns	0,4843
RochM	Untreated control vs. 5000	Yes	****	< 0,0001
	Untreated control vs. 2500	Yes	****	< 0,0001
	Untreated control vs. 1250	No	ns	0,1325

Legend: * Concentration unit of all extracts is [mg/L]; ns – not significant.
