# Supplementary material for: Outstanding Antibacterial Activity of Hypericum rochelii—Comparison of the Antimicrobial Effects of Extracts and Fractions from Four Hypericum Species Growing in Bulgaria with a Focus on Prenylated Phloroglucinols
Source: Life (Basel). 2023 Jan 18;13(2):274. doi: 10.3390/life13020274 (PMC9959064; doi:10.3390/life13020274)
Supplement: Supplementary file 1 [file life-13-00274-s001.zip › life-1975708-supplementary/Suppl. Table S4 DEHA E. facalis statistics.pdf]

Table S4. One-way ANOVA of the metabolic activity of *Enterococcus faecalis*. Comparison between the treated groups and untreated control.

Extract	Dunnett's multiple comparisons test	Significance		Adjusted P Value
RochC	Untreated control vs. 1250 *	Yes	****	< 0,0001
	Untreated control vs. 625	Yes	****	< 0,0001
	Untreated control vs. 313	Yes	****	< 0,0001
	Untreated control vs. 156	Yes	****	< 0,0001
	Untreated control vs. 78	Yes	****	< 0,0001
	Untreated control vs. 39	Yes	****	< 0,0001
	Untreated control vs. 19,5	Yes	****	< 0,0001
	Untreated control vs. 9,8	Yes	****	< 0,0001
	Untreated control vs. 4,9	Yes	****	< 0,0001
	Untreated control vs. 2,5	Yes	***	0,0001
HirDM90	Untreated control vs. 5000	Yes	****	< 0,0001
	Untreated control vs. 2500	Yes	****	< 0,0001
	Untreated control vs. 1250	Yes	****	< 0,0001
	Untreated control vs. 625	Yes	****	< 0,0001
	Untreated control vs. 313	Yes	****	< 0,0001
	Untreated control vs. 156	Yes	****	< 0,0001
	Untreated control vs. 78	Yes	****	< 0,0001
	Untreated control vs. 39	Yes	****	< 0,0001
	Untreated control vs. 19,5	Yes	****	< 0,0001
	Untreated control vs. 9,8	Yes	****	< 0,0001
RochD	Untreated control vs. 5000	Yes	****	< 0,0001
	Untreated control vs. 2500	Yes	****	< 0,0001
	Untreated control vs. 1250	Yes	****	< 0,0001
	Untreated control vs. 625	Yes	****	< 0,0001
	Untreated control vs. 313	Yes	****	< 0,0001
	Untreated control vs. 156	Yes	*	0,0458
	Untreated control vs. 78	No	ns	0,0544
	Untreated control vs. 39	Yes	*	0,0117
	Untreated control vs. 19,5	No	ns	0,6840
	Untreated control vs. 9,8	No	ns	0,0506
	Untreated control vs. 4,9	Yes	*	0,0129
RochCM	Untreated control vs. 5000	Yes	****	< 0,0001
	Untreated control vs. 2500	Yes	****	< 0,0001
	Untreated control vs. 1250	Yes	****	< 0,0001
	Untreated control vs. 625	Yes	****	< 0,0001
	Untreated control vs. 313	Yes	****	< 0,0001
	Untreated control vs. 156	Yes	****	< 0,0001
	Untreated control vs. 78	Yes	****	< 0,0001
	Untreated control vs. 39	Yes	**	0,0048
	Untreated control vs. 19,5	Yes	**	0,0024
	Untreated control vs. 9,8	Yes	***	0,0006
RumDBe	Untreated control vs. 5000	Yes	*	0,0391

Legend: * Concentrations of the extract in [mg/L]; ns – not significant.
