# Supplementary material for: Outstanding Antibacterial Activity of Hypericum rochelii—Comparison of the Antimicrobial Effects of Extracts and Fractions from Four Hypericum Species Growing in Bulgaria with a Focus on Prenylated Phloroglucinols
Source: Life (Basel). 2023 Jan 18;13(2):274. doi: 10.3390/life13020274 (PMC9959064; doi:10.3390/life13020274)
Supplement: Supplementary file 1 [file life-13-00274-s001.zip › life-1975708-supplementary/Suppl. Table S6 DEHA E. coli statistics.pdf]

Table S6. One-way ANOVA of the metabolic activity of *Escherichia coli*. Comparison between the treated groups and untreated control.

Extract	Dunnett's multiple comparisons test	Significance		Adjusted P Value
RochC	Untreated control vs. 5000 *	Yes	****	< 0,0001
	Untreated control vs. 2500	Yes	****	< 0,0001
	Untreated control vs. 1250	Yes	****	< 0,0001
	Untreated control vs. 625	Yes	****	< 0,0001
	Untreated control vs. 313	Yes	****	< 0,0001
	Untreated control vs. 156	Yes	****	< 0,0001
	Untreated control vs. 78	Yes	****	< 0,0001
	Untreated control vs. 39	Yes	****	< 0,0001
HirDM90	Untreated control vs. 5000	Yes	****	< 0,0001
	Untreated control vs. 2500	Yes	****	< 0,0001
	Untreated control vs. 1250	Yes	****	< 0,0001
	Untreated control vs. 625	Yes	****	< 0,0001
	Untreated control vs. 313	Yes	***	0,0003
RochD	Untreated control vs. 5000	Yes	***	0,0001
	Untreated control vs. 2500	Yes	***	0,0003
	Untreated control vs. 1250	Yes	**	0,0073
	Untreated control vs. 625	No	ns	0,3216
	Untreated control vs. 313	Yes	***	0,0001
RochCM	Untreated control vs. 5000	Yes	****	< 0,0001
	Untreated control vs. 2500	Yes	****	< 0,0001
	Untreated control vs. 1250	Yes	****	< 0,0001
	Untreated control vs. 625	Yes	****	< 0,0001
	Untreated control vs. 313	Yes	****	< 0,0001
	Untreated control vs. 156	Yes	****	< 0,0001
	Untreated control vs. 78	Yes	**	0,0051
	Untreated control vs. 39	Yes	****	< 0,0001
BarbD	Untreated control vs. 5000	Yes	**	0,0042
	Untreated control vs. 2500	Yes	**	0,0065
RumDBe	Untreated control vs. 5000	No	ns	0,0528
	Untreated control vs. 2500	No	ns	0,0897
RochM	Untreated control vs. 5000	Yes	**	0,0015
	Untreated control vs. 2500	Yes	**	0,0037
	Untreated control vs. 1250	Yes	*	0,0178

Legend: * Concentrations of the extract in [mg/L]; ns – not significant.
