# Supplementary material for: Outstanding Antibacterial Activity of Hypericum rochelii—Comparison of the Antimicrobial Effects of Extracts and Fractions from Four Hypericum Species Growing in Bulgaria with a Focus on Prenylated Phloroglucinols
Source: Life (Basel). 2023 Jan 18;13(2):274. doi: 10.3390/life13020274 (PMC9959064; doi:10.3390/life13020274)
Supplement: Supplementary file 1 [file life-13-00274-s001.zip › life-1975708-supplementary/Suppl. Table S7 DEHA P. aeruginosa statistics.pdf]

Table S7. One-way ANOVA of the metabolic activity of *Pseudomonas aeruginosa*. Comparison between the treated groups and untreated control.

Extract	Dunnett's multiple comparisons test	Significance		Adjusted P Value
RochC	Untreated control vs. 5000 *	Yes	****	< 0,0001
	Untreated control vs. 2500	Yes	****	< 0,0001
	Untreated control vs. 1250	Yes	****	< 0,0001
	Untreated control vs. 625	Yes	***	0,0003
	Untreated control vs. 313	Yes	**	0,0060
	Untreated control vs. 156	Yes	**	0,0057
	Untreated control vs. 78	Yes	*	0,0479
	Untreated control vs. 39	No	ns	0,9996
HirDM90	Untreated control vs. 5000	Yes	***	0,0003
	Untreated control vs. 2500	Yes	*	0,0109
RochD	Untreated control vs. 5000	Yes	****	< 0,0001
	Untreated control vs. 2500	Yes	****	< 0,0001
	Untreated control vs. 1250	Yes	****	< 0,0001
	Untreated control vs. 625	Yes	****	< 0,0001
	Untreated control vs. 313	Yes	****	< 0,0001
	Untreated control vs. 156	Yes	*	0,0310
	Untreated control vs. 78	Yes	***	0,0003
	Untreated control vs. 39	Yes	***	0,0002
	Untreated control vs. 19,5	No	ns	0,0613
RochCM	Untreated control vs. 5000	Yes	****	< 0,0001
	Untreated control vs. 2500	Yes	***	0,0003
	Untreated control vs. 1250	Yes	***	0,0005
	Untreated control vs. 625	No	ns	0,2078
	Untreated control vs. 313	No	ns	0,5679
	Untreated control vs. 156	No	ns	0,9994
	Untreated control vs. 78	No	ns	0,9995
	Untreated control vs. 39	No	ns	0,8420
BarbD	Untreated control vs. 5000	Yes	***	0,0003
	Untreated control vs. 2500	Yes	**	0,0025
	Untreated control vs. 1250	Yes	**	0,0038
HirDD	Untreated control vs. 5000	No	ns	0,8013

Legend: * Concentrations of the extract in [mg/L]; ns – not significant.
