# Supplementary material for: Outstanding Antibacterial Activity of Hypericum rochelii—Comparison of the Antimicrobial Effects of Extracts and Fractions from Four Hypericum Species Growing in Bulgaria with a Focus on Prenylated Phloroglucinols
Source: Life (Basel). 2023 Jan 18;13(2):274. doi: 10.3390/life13020274 (PMC9959064; doi:10.3390/life13020274)
Supplement: Supplementary file 1 [file life-13-00274-s001.zip › life-1975708-supplementary/Suppl. Table S8 DEHA Y. enterocolitica statistics.pdf]

Table S8. One-way ANOVA of the metabolic activity of *Yersinia enterocolitica*. Comparison between the treated groups and untreated control.

Extract	Dunnett's multiple comparisons test	Significance		Adjusted P Value
RochC	Untreated control vs. 5000 *	Yes	*	0,0147
	Untreated control vs. 2500	Yes	*	0,0497
RochCM	Untreated control vs. 5000	No	ns	0,9998

Legend: * Concentrations of the extract in [mg/L]; CI – confidence interval; ns – not significant.
