# Supplementary material for: Outstanding Antibacterial Activity of Hypericum rochelii—Comparison of the Antimicrobial Effects of Extracts and Fractions from Four Hypericum Species Growing in Bulgaria with a Focus on Prenylated Phloroglucinols
Source: Life (Basel). 2023 Jan 18;13(2):274. doi: 10.3390/life13020274 (PMC9959064; doi:10.3390/life13020274)
Supplement: Supplementary file 1 [file life-13-00274-s001.zip › life-1975708-supplementary/Suppl. Tables S9,10 Biofilm absorbance ANOVA.pdf]

Table S9. One-way ANOVA of the biofilm absorbance. Column statistics.

**Extract**	**RochD**				
Concentrations*	Untreated control	0,625	1,25	2,5	
Mean	1,331	0,4280	0,2615	0,2525	
Std. Deviation	0,09899	0,03111	0,05586	0,01768	
**Extract**	**HirDM90**				
Concentrations	Untreated control	2,06	4,125	8,25	16,5
Mean	1,331	0,3410	0,3145	0,2555	0,2505
Std. Deviation	0,09899	0,01273	0,007778	0,007778	0,09970
**Extract**	**HirDM100**				
Concentrations	Untreated control	9,53125	19,0625	38,125	
Mean	1,331	0,3230	0,3115	0,2875	
Std. Deviation	0,09899	0,05233	0,04313	0,06293	
**Extract**	**RumDKo**				
Concentrations	Untreated control	312,5	625	1250	2500
Mean	0,7115	0,6440	0,6140	0,1890	0,0700
Std. Deviation	0,006364	0,002828	0,002828	0,04384	0,009899

Legend: * Concentration unit is [mg/L].

Table S10. One-way ANOVA of the biofilm absorbance. Comparison between the treated groups and untreated control.

**Extract**	**Dunnett's multiple comparisons test**	**Mean Difference**	**95% CI of difference**	**Significant?**	**Summary**	**Adjusted P Value**
RochD	Untreated control vs. 0,625*	0,903	0,618 to 1,19	Yes	**	0,0019
	Untreated control vs. 1,25	1,07	0,784 to 1,35	Yes	**	0,0011
	Untreated control vs. 2,5	1,08	0,793 to 1,36	Yes	**	0,0011
HirDM90	Untreated control vs. 2,06	0,990	0,777 to 1,20	Yes	***	0,0002
	Untreated control vs. 4,125	1,02	0,803 to 1,23	Yes	***	0,0002
	Untreated control vs. 8,25	1,08	0,862 to 1,29	Yes	***	0,0001
	Untreated control vs. 16,5	1,08	0,867 to 1,29	Yes	***	0,0001
HirDM100	Untreated control vs. 9,53125	1,01	0,904 to 1,11	Yes	****	< 0,0001
	Untreated control vs. 19,0625	1,02	0,915 to 1,12	Yes	****	< 0,0001
	Untreated control vs. 38,125	1,04	0,939 to 1,15	Yes	****	< 0,0001
RumDKo	Untreated control vs. 312,5	0,0675	-0,0127 to 0,148	No	ns	0,0847
	Untreated control vs. 625	0,0975	0,0173 to 0,178	Yes	*	0,0262
	Untreated control vs. 1250	0,523	0,442 to 0,603	Yes	****	< 0,0001
	Untreated control vs. 2500	0,642	0,561 to 0,722	Yes	****	< 0,0001

Legend: * Concentrations of the extract in [mg/L].
