# Supplementary material for: Outstanding Antibacterial Activity of Hypericum rochelii—Comparison of the Antimicrobial Effects of Extracts and Fractions from Four Hypericum Species Growing in Bulgaria with a Focus on Prenylated Phloroglucinols
Source: Life (Basel). 2023 Jan 18;13(2):274. doi: 10.3390/life13020274 (PMC9959064; doi:10.3390/life13020274)
Supplement: Supplementary file 1 [file life-13-00274-s001.zip › life-1975708-supplementary/Supplementary figures S1-3.pdf]

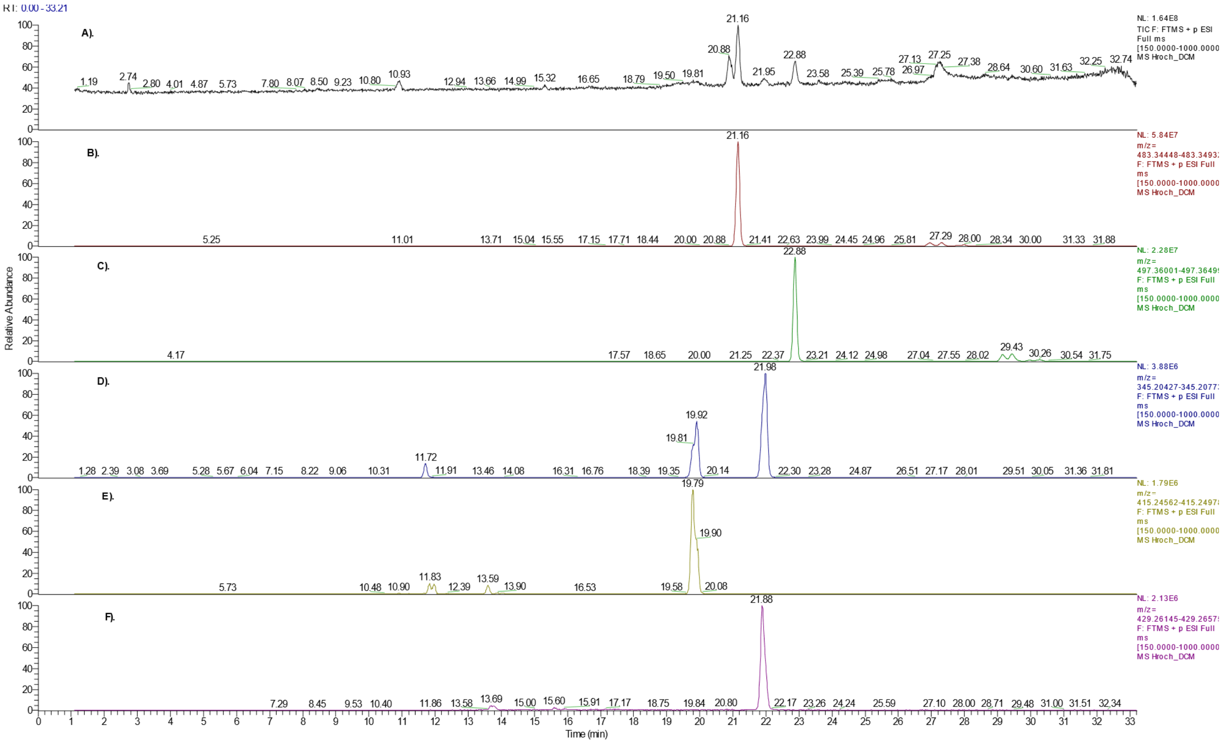

Figure S1. Chromatographic profile of RochD. A). Total Ion Chromatogram (TIC); B). eXtracted Ion Chromatogram (XIC) at *m/z* 483.3469; C). XIC at *m/z* 497.3625; D). XIC at m/z 345.2060; E). XIC at *m/z* 415.2477; F). XIC at *m/z* 429.2636.

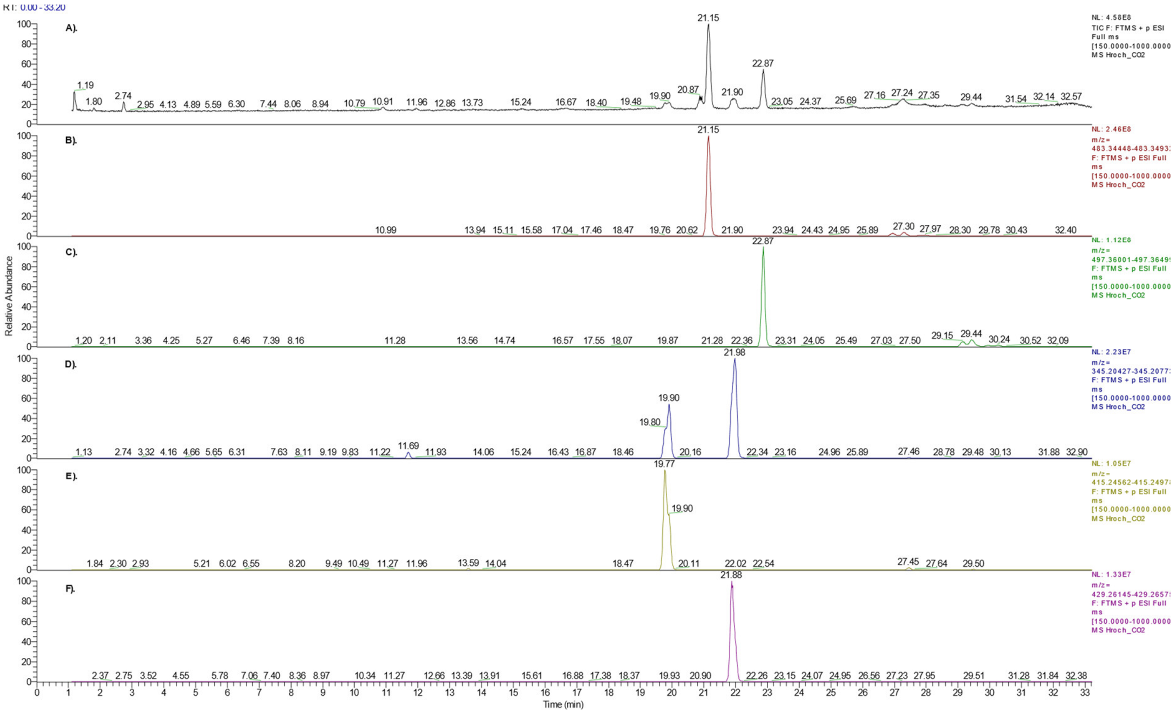

Figure S2. Chromatographic profile of RochC. A). TIC; B). XIC at *m/z* 483.3469; C). XIC at *m/z* 497.3625; D). XIC at *m/z* 345.2060; E). XIC at *m/z* 415.2477; F). XIC at *m/z* 429.2636.

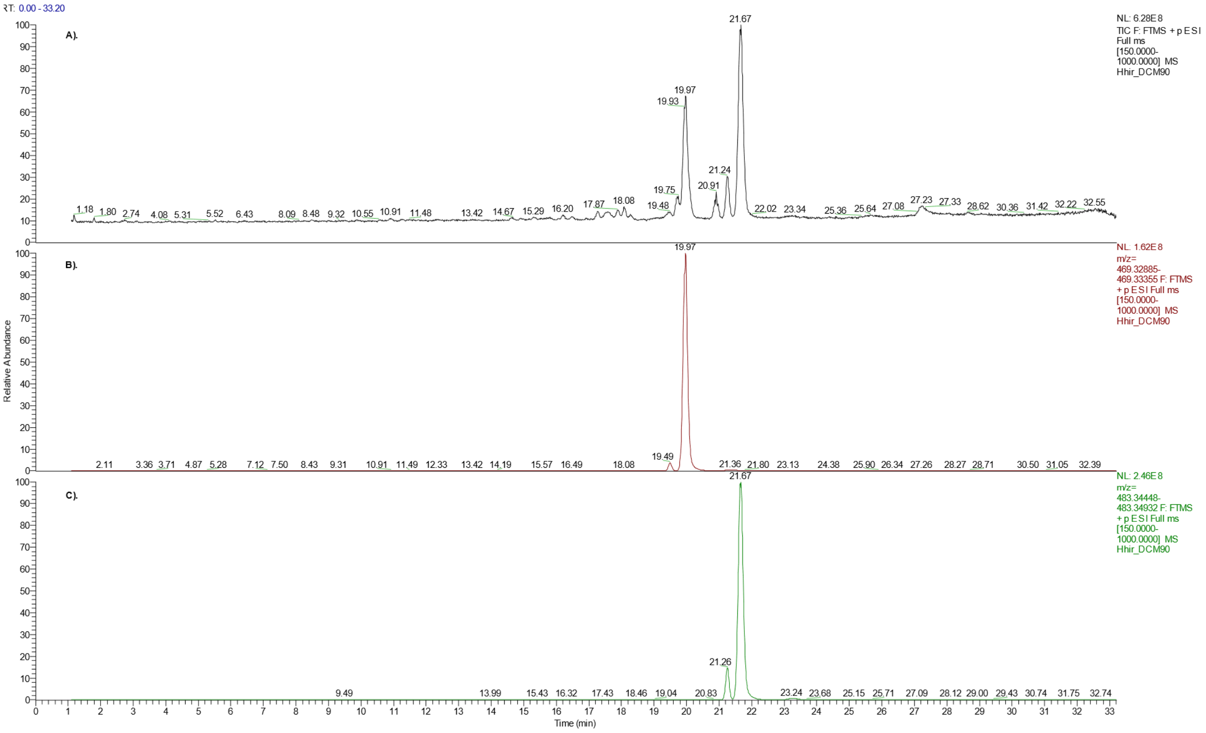

Figure S3. Chromatographic profile of HirDM90. A). TIC; B). XIC at *m/z* 469.3312; C). XIC at *m/z* 483.3469.
