# Supplementary material for: Outstanding Antibacterial Activity of Hypericum rochelii—Comparison of the Antimicrobial Effects of Extracts and Fractions from Four Hypericum Species Growing in Bulgaria with a Focus on Prenylated Phloroglucinols
Source: Life (Basel). 2023 Jan 18;13(2):274. doi: 10.3390/life13020274 (PMC9959064; doi:10.3390/life13020274)
Supplement: Supplementary file 1 [file life-13-00274-s001.zip › life-1975708-supplementary/Table S1 Means STDEV Metab. Activity.pdf]

Table S1. One-way ANOVA of the metabolic activity. Column statistics.

		**Bacterial strains**														
**Extracts**		**A.** ***Staphylococcus aureus***														
**RochC**	Concentrations [mg/L]	Untreated control	1250	625	313	156	78	39	19,5	9,8	4,9	2,5	1,23	0,61		
	Mean [%]	100,0	0,0	2,844	0,0	0,0	0,0	0,0	0,0	0,0	2,369	2,811	1,807	73,00		
	Std. deviation	±10,27	±1,027	±4,917	±1,541	±1,130	±1,315	±0,9607	±1,469	±1,593	±0,7382	±1,022	±0,8518	±1,136		
**HirDM90**	Concentrations [mg/L]	Untreated control	5000	±	1250	625	313	156	78	39	19,5	9,8	4,9	2,5	1,23	0,61
	Mean [%]	100,0	3,332	3,002	2,589	1,405	0,5784	0,3305	0,4957	0,3856	1,184	0,6059	0,8432	1,847	1,325	92,99
	Std. deviation	±6,232	±0,4284	±0,5063	±0,3895	±0,4284	±0,4284	±0,07790	±0,3895	±0,1558	±0,6621	±0,07790	±1,647	±0,4543	±0,6246	±9,199
**RochD**	Concentrations [mg/L]	Untreated control	5000	2500	1250	625	313	156	78	39	19,5	9,8	4,9			
	Mean [%]	100,0	0,0	0,0	0,0	0,0	0,0	4,046	3,463	2,171	1,984	1,692	1,047			
	Std. deviation	±11,78	±3,829	±5,066	±1,649	±0,6480	±2,297	±0,1767	±0,1767	±0,1767	±0,3829	±0,8542	±0,3535			
**RochCM**	Concentrations [mg/L]	Untreated control	5000	2500	1250	625	313	156	78	39	19,5	9,8	4,9	2,5		
	Mean [%]	100,0	0,0	0,0	0,0	0,0	0,0	0,0	0,0	0,0	0,0	0,0	0,9637	32,04		
	Std. deviation	±10,27	±5,959	±3,288	±4,829	±6,370	±1,952	±4,418	±2,980	±1,130	±3,802	±5,651	±0,4543	±5,906		
**BarbD**	Concentrations [mg/L]	Untreated control	5000	2500	1250	625	313	156								
	Mean [%]	100,0	0,0	0,0	0,0	33,90	48,31	81,62								
	Std. deviation	±15,63	±0,4775	±1,117	±0,9323	±7,107	±2,770	±1,929								
**HirDM100**	Concentrations [mg/L]	Untreated control	5000	2500	1250	625	313	156	78	39	19,5	9,8				
	Mean [%]	100,0	14,49	8,014	4,682	3,332	3,305	2,286	5,150	4,517	5,067	8,014				
	Std. deviation	±6,232	±4,907	±1,597	±0,8569	±1,441	±2,571	±2,142	±5,648	±5,453	±5,686	±7,751				
**RumDBe**	Concentrations [mg/L]	Untreated control	5000	2500	1250	625										
	Mean [%]	100,0	0,2623	0,0	0,0	32,69										
	Std. deviation	±11,05	±1,271	±0,01658	±0,02763	±0,7183										
**RumDKo**	Concentrations [mg/L]	Untreated control	5000	2500	1250	625										
	Mean [%]	100,0	1,838	10,10	82,74	100,0										
	Std. deviation	±15,63	±0,7427	±7,051	±10,25	±15,63										
**RochM**	Concentrations [mg/L]	Untreated control	5000	2500	1250											
	Mean [%]	100,0	7,554	12,07	79,20											
	Std. deviation	±5,891	±5,596	±5,596	±5,596											
**HirrD**	Concentrations	Untreated	5000													

	[mg/L]	control											
	Mean [%]	100,0	79,87										
	Std. deviation	±6,232	±7,244										
							**B. MRSA**						
**RochC**	Concentrations [mg/L]	Untreated control	5000	2500	1250	625	313	156	78	39	19,5	9,8	
	Mean [%]	100,0	1,451	1,451	1,451	1,451	1,451	1,451	1,451	1,451	1,451	1,451	
	Std. deviation	±1,845	±5,350	±8,486	±1,015	±3,321	±5,258	±7,656	±2,583	±9,132	±3,782	±7,103	
**HirDM90**	Concentrations [mg/L]	Untreated control	5000	2500	1250	625	313	156	78	39	19,5	9,8	
	Mean [%]	100,0	5,105	7,707	4,761	1,129	0,0	0,0	0,0	3,092	41,53	81,58	
	Std. deviation	±13,88	±1,666	±0,3471	±0,3471	±0,06942	±0,1388	±0,3471	±0,3471	±6,178	±2,221	±7,081	
**RochD**	Concentrations [mg/L]	Untreated control	5000	2500	1250	625	313	156	78	39	19,5	9,8	4,9
	Mean [%]	100,0	0,0	0,0	0,0	0,0	0,0	4,046	3,463	2,171	1,984	1,692	1,047
	Std. deviation	±11,78	±2,474	±1,655	±5,066	±0,8837	±1,944	±0,1767	±0,1767	±0,1767	±0,3829	±0,8542	±0,3535
**RochCM**	Concentrations [mg/L]	Untreated control	5000	2500	1250	625	313	156	78	39	19,5	9,8	
	Mean [%]	100,0	5,267	5,267	5,267	5,267	5,267	5,267	30,12	69,87	92,28	86,41	
	Std. deviation	±1,845	±10,15	±8,486	±12,36	±1,845	±7,195	±0,6457	±6,549	±3,367	±1,660	±2,491	
**BarbD**	Concentrations [mg/L]	Untreated control	5000	2500	1250	625	313	156	78				
	Mean [%]	100,0	0,0	6,193	31,58	57,29	89,49	79,02	88,51				
	Std. deviation	±1,682	±0,4204	±1,934	±5,970	±0,4624	±0,5886	±1,934	±3,489				
**RumDBe**	Concentrations [mg/L]	Untreated control	5000	2500	1250	625	313	156	78				
	Mean [%]	100,0	0,0	0,6936	14,84	34,31	49,80	58,51	88,18				
	Std. deviation	±1,682	±0,4204	±1,135	±0,2102	±1,093	±3,405	±1,682	±1,598				
**RumDKo**	Concentrations [mg/L]	Untreated control	5000	2500	1250	625	313	156					
	Mean [%]	100,0	0,0	1,823	23,02	48,67	59,20	82,65					
	Std. deviation	±1,682	±1,682	±0,3784	±2,775	±2,817	±3,657	±8,576					
							**C. *Enterococcus faecalis***						
**RochC**	Concentrations [mg/L]	Untreated control	1250	625	313	156	78	39	19,5	9,8	4,9	2,5	
	Mean [%]	100,0	26,25	17,28	8,415	3,670	0,7414	0,0	0,5561	0,7414	4,523	47,82	
	Std. deviation	±10,49	±3,408	±0,1573	±1,153	±0,6291	±0,7864	±0,7864	±0,8388	±0,6815	±1,625	±1,311	
**HirDM90**	Concentrations	Untreated	5000	2500	1250	625	313	156	78,125	39,0625	19,5	9,8	

	[mg/L]	control											
	Mean [%]	100,0	9,210	11,44	8,032	5,675	2,977	2,202	1,861	5,055	41,18	70,80	
	Std. deviation	±8,771	±1,973	±0,04385	±0,1316	±0,1316	±0,4386	±0,1316	±0,4386	±0,4824	±7,192	±1,623	
**RochD**	Concentrations [mg/L]	Untreated control	5000	2500	1250	625	313	156	78	39	19,5	9,8	4,9
	Mean [%]	100,0	8,706	8,871	9,199	25,05	3,121	68,75	69,73	60,86	86,49	69,32	61,44
	Std. deviation	±9,293	±1,859	±10,92	±2,672	±5,808	±1,742	±3,949	±10,45	±9,293	±1,859	±6,853	±4,530
**RochCM**	Concentrations [mg/L]	Untreated control	5000	2500	1250	625	313	156	78	39	19,5	9,8	
	Mean [%]	100,0	1,779	1,779	1,779	1,779	1,779	1,779	14,16	58,50	54,38	45,38	
	Std. deviation	±10,49	±6,082	±1,258	±10,49	±3,670	±5,033	±10,07	±2,569	±0,7864	±4,404	±0,6815	
**RumDBe**	Concentrations [mg/L]	Untreated control	5000										
	Mean [%]	100,0	38,63										
	Std. deviation	±10,91	±13,53										

D. ***Streptococcus pyogenes***

**RochC**	Concentrations [mg/L]	Untreated control	5000	2500	1250	625	313	156	78	39	19,5	9,8
	Mean [%]	100,0	0,0	1,380	1,380	1,380	1,380	1,380	0,0	0,0	0,0	0,0
	Std. deviation	±4,762	±4,762	±4,095	±12,19	±1,048	±5,047	±1,762	±1,286	±0,9047	±1,286	±0,6666
**HirDM90**	Concentrations [mg/L]	Untreated control	5000	2500	1250	625	313	156	78	39	19,5	9,8
	Mean [%]	100,0	8,252	12,72	10,02	5,851	4,014	3,001	2,476	28,69	59,15	81,21
	Std. deviation	±5,305	±0,1061	±1,220	±0,6896	±0,1061	±3,554	±0,9548	±0,2122	±2,387	±0,7957	±0,9018
**RochD**	Concentrations [mg/L]	Untreated control	5000	2500	1250	625	313	156	78	39	19,5	9,8
	Mean [%]	100,0	-3,599	-3,013	-2,916	-3,111	-2,525	12,16	11,38	63,94	76,43	96,63
	Std. deviation	±13,80	±1,104	±1,104	±1,104	±1,104	±0,4141	±0,2070	±1,449	±5,797	±0,8281	±0,0
**RochCM**	Concentrations [mg/L]	Untreated control	5000	2500	1250	625	313	156	78	39	19,5	9,8
	Mean [%]	100,0	6,902	6,902	6,902	6,902	6,902	9,798	18,82	34,88	51,68	66,43
	Std. deviation	±4,762	±8,285	±8,571	±3,333	±12,09	±0,9047	±0,04762	±0,4286	±2,762	±2,428	±2,905
**BarbD**	Concentrations [mg/L]	Untreated control	5000	2500	1250	625	313	156				
	Mean [%]	100,0	30,90	38,42	46,73	52,85	62,15	74,93				
	Std. deviation	±3,658	±3,904	±2,558	±10,87	±4,964	±5,074	±1,245				
**HirDM100**	Concentrations [mg/L]	Untreated control	5000	2500	1250							
	Mean [%]	100,0	66,84	68,49	84,77							

	Std. deviation	±5,305	±7,214	±11,99	±0,2122						
**RumDBe**	Concentrations [mg/L]	Untreated control	5000	2500	1250	625	313	156			
	Mean [%]	100,0	5,661	8,003	35,73	50,66	54,68	70,12			
	Std. deviation	±5,174	±2,483	±3,104	±2,483	±2,483	±8,071	±1,966			
**RumDKo**	Concentrations [mg/L]	Untreated control	5000	2500	1250	625	313	156	78	39	
	Mean [%]	100,0	10,76	51,66	55,73	60,63	80,63	84,85	88,19	97,39	
	Std. deviation	±3,658	±4,810	±6,167	±4,756	±6,676	±8,589	±6,684	±9,040	±2,579	

E. *Escherichia coli*

**RochC**	Concentrations [mg/L]	Untreated control	Untreated control	5000	2500	1250	625	313	156	78	39
	Mean [%]	100,0	100,0	17,02	23,54	18,66	42,02	48,50	59,63	66,15	81,60
	Std. deviation	±2,890	±2,890	±2,572	±2,312	±4,538	±3,873	±2,803	±3,988	±5,462	±0,4335
**HirDM90**	Concentrations	Untreated control	5000	2500	1250	625	313				
	Mean [%]	100,0	6,199	11,13	27,00	73,91	81,61				
	Std. deviation	±3,211	±0,3532	±1,991	±5,138	±3,211	±3,211				
**RochD**	Concentrations [mg/L]	Untreated control	5000	2500	1250	625					
	Mean [%]	100,0	20,32	36,44	73,17	92,48					
	Std. deviation	±2,907	±5,233	±2,791	±3,227	±4,651					
**RochCM**	Concentrations [mg/L]	Untreated control	5000	2500	1250	625	313	156	78	39	
	Mean [%]	100,0	36,44	35,95	54,91	66,89	68,54	71,61	86,28	74,41	
	Std. deviation	±2,312	±0,08671	±1,012	±3,613	±1,416	±2,659	±5,202	±1,561	±1,243	
**BarbD**	Concentrations [mg/L]	Untreated control	5000	2500							
	Mean [%]	100,0	76,35	79,01							
	Std. deviation	±3,232	±2,457	±4,441							
**RumDBe**	Concentrations [mg/L]	Untreated control	5000	2500							
	Mean [%]	100,0	87,57	90,67							
	Std. deviation	±4,570	±1,371	±5,979							
**RochM**	Concentrations [mg/L]	Untreated control	5000	2500	1250						
	Mean [%]	100,0	70,50	78,35	87,50						
	Std. deviation	±2,907	±1,948	±0,9012	±1,047						

F. *Pseudomonas aeruginosa*

**RochC**	Concentrations [mg/L]	Untreated control	1250	625	313	156	78	39			
	Mean [%]	100,0	16,43	27,16	29,19	46,08	65,22	64,90			
	Std. deviation	±2,606	±2,411	±2,997	±3,258	±0,6842	±8,145	±11,34			
**HirDM90**	Concentrations [mg/L]	Untreated control	5000	2500							
	Mean [%]	100,0	7,799	42,25							
	Std. deviation	±4,258	±0,8943	±14,86							
**RochD**	Concentrations [mg/L]	Untreated control	5000	2500	1250	625	313	156	78	39	19
	Mean [%]	100,0	-3,019	-2,002	4,400	27,38	49,25	81,81	65,72	64,11	83,93
	Std. deviation	±2,998	±0,0	±0,4197	±2,038	±7,315	±3,358	±1,979	±8,724	±4,407	±1,739
**RochCM**	Concentrations [mg/L]	Untreated control	5000	2500	1250	625	313				
	Mean [%]	100,0	40,36	54,88	57,41	86,32	91,09				
	Std. deviation	±2,606	±8,178	±2,769	±1,727	±5,278	±0,2281				
**BarbD**	Concentrations [mg/L]	Untreated control	5000	2500	1250						
	Mean [%]	100,0	44,95	58,43	60,75						
	Std. deviation	±3,531	±1,765	±7,132	±0,6708						
**HirDD**	Concentrations [mg/L]	Untreated control	5000								
	Mean [%]	100,0	91,39								
	Std. deviation	±4,258	±0,4684								

G. *Yersinia enterocolitica*

**RochC**	Concentrations [mg/L]	Untreated control	5000	2500
	Mean [%]	100,0	43,22	60,36
	Std. deviation	±5,103	±9,899	±15,87
**RochCM**	Concentrations [mg/L]	Untreated control	5000	
	Mean [%]	100,0	94,31	
	Std. deviation	±5,103	±6,837	
